# Multivariate Protein Signatures of Pre-Clinical Alzheimer's Disease in the Alzheimer's Disease Neuroimaging Initiative (ADNI) Plasma Proteome Dataset

**DOI:** 10.1371/journal.pone.0034341

**Published:** 2012-04-02

**Authors:** Daniel Johnstone, Elizabeth A. Milward, Regina Berretta, Pablo Moscato, for the Alzheimer's Disease Neuroimaging Initiative

**Affiliations:** 1 Priority Research Centre for Bioinformatics, Biomarker Discovery and Information-Based Medicine, The University of Newcastle, Callaghan, New South Wales, Australia; 2 School of Electrical Engineering and Computer Science, The University of Newcastle, Callaghan, New South Wales, Australia; 3 School of Biomedical Sciences and Pharmacy, The University of Newcastle, Callaghan, New South Wales, Australia; Massachusetts General Hospital and Harvard Medical School, United States of America

## Abstract

**Background:**

Recent Alzheimer's disease (AD) research has focused on finding biomarkers to identify disease at the pre-clinical stage of mild cognitive impairment (MCI), allowing treatment to be initiated before irreversible damage occurs. Many studies have examined brain imaging or cerebrospinal fluid but there is also growing interest in blood biomarkers. The Alzheimer's Disease Neuroimaging Initiative (ADNI) has generated data on 190 plasma analytes in 566 individuals with MCI, AD or normal cognition. We conducted independent analyses of this dataset to identify plasma protein signatures predicting pre-clinical AD.

**Methods and Findings:**

We focused on identifying signatures that discriminate cognitively normal controls (*n* = 54) from individuals with MCI who subsequently progress to AD (*n* = 163). Based on *p* value, apolipoprotein E (APOE) showed the strongest difference between these groups (*p* = 2.3×10^−13^). We applied a multivariate approach based on combinatorial optimization (*(α,β)-k Feature Set Selection*), which retains information about individual participants and maintains the context of interrelationships between different analytes, to identify the optimal set of analytes (signature) to discriminate these two groups. We identified 11-analyte signatures achieving values of sensitivity and specificity between 65% and 86% for both MCI and AD groups, depending on whether APOE was included and other factors. Classification accuracy was improved by considering “meta-features,” representing the difference in relative abundance of two analytes, with an 8-meta-feature signature consistently achieving sensitivity and specificity both over 85%. Generating signatures based on longitudinal rather than cross-sectional data further improved classification accuracy, returning sensitivities and specificities of approximately 90%.

**Conclusions:**

Applying these novel analysis approaches to the powerful and well-characterized ADNI dataset has identified sets of plasma biomarkers for pre-clinical AD. While studies of independent test sets are required to validate the signatures, these analyses provide a starting point for developing a cost-effective and minimally invasive test capable of diagnosing AD in its pre-clinical stages.

## Introduction

Alzheimer's disease (AD) is a progressive, fatal neurodegenerative disorder characterized by memory loss and other cognitive impairments. There is currently no known cure, with treatments generally aimed at slowing disease progression. Beneficial outcomes from treatment rely on identifying disease at its early stages (e.g. when only mild cognitive impairment is present), making timely, accurate forms of early diagnosis essential (reviewed in [1]–[3]). As a result there has been a great deal of recent research activity aimed at identifying diagnostic biomarkers of pre-clinical AD.

From this research it is becoming increasingly apparent that univariate biomarkers are not sufficiently sensitive or specific for the diagnosis of complex, multifactorial disorders such as AD [4]. Instead researchers will need to consider applying multivariate approaches in order to identify reliable biomarker signatures of pre-clinical AD. The most consistent and reliable biomarkers of AD identified to date require expensive imaging procedures or invasive collection of cerebrospinal fluid (CSF) [5], [6], whereas ideal biomarkers would be measurable using cost-effective and minimally-invasive techniques [7], [8].

This has led many groups to investigate the diagnostic potential of signatures based on protein levels in blood plasma. In 2007 a study by Ray and colleagues, published in *Nature Medicine* [9], proposed that the abundance of 18 proteins in plasma can predict the onset of clinical AD between two and six years before the disease clearly manifests. These proteins were identified from a larger panel of growth factors, cytokines and other immune response proteins. On two test sets of samples from 42 individuals with clinical AD and 47 with mild cognitive impairment (MCI), which can progress to AD, this 18-protein signature was able to correctly classify 81% of AD patients and accurately predict progression from MCI to AD in 91% of individuals assessed [9], raising hopes that a blood-based test for AD may soon be within reach.

Using a different mathematical approach, our group demonstrated in 2008 that a set of only five proteins (tumor necrosis factor-α, interleukin-1α, interleukin-3, granulocyte colony stimulating factor, epidermal growth factor) from the original 18–protein signature achieves an even better prediction accuracy within the restricted confines of the Ray dataset, with 100% sensitivity in predicting AD and 92% specificity in identifying cognitively normal controls [10].

However the study of Ray and colleagues has various limitations. Data were generated from membrane-based arrays exposed to autoradiographic film, an assay method that is not commonly used and has not been widely validated for quantitative studies. In addition, the dataset that was presented in the paper comprised Z-score transformed spot intensities rather than protein concentration data calculated through the use of standard curves, making it difficult to assess absolute protein signals relative to background. Possibly as a consequence of this, other groups have not been able to replicate the findings of original study by Ray and colleagues [7], [11], [12], with several of the 18 proteins in the signature being shown to have plasma levels below the detection limits of other assays [12].

Recently the Alzheimer's Disease Neuroimaging Initiative (ADNI), a large collaborative project aimed at better understanding MCI and AD, made its data available to the scientific community. These data include various clinical, cognitive, imaging and genetic measures, as well as the ‘Plasma Proteome Dataset’. The Plasma Proteome Dataset, generated using a bead-based multiplexing assay on the Luminex platform, comprises data on levels of 190 analytes in plasma from cognitively normal controls, individuals with MCI and AD patients. This dataset represents a substantial improvement on the previous public domain datasets, with a larger sample size, accessible raw data and detailed documentation relating to the experimental procedures, quality control measures and data analyses.

Our research group has been pioneering the use of multivariate approaches based on combinatorial optimization to identify molecular signatures of different diseases, including plasma protein signatures of AD [10], [13]. In contrast to routine statistical methods that meld individual data to generate group summary values, these approaches retain information about individual participants and thereby also preserve the context of interrelationships between different analytes. In this study, we have applied our analytical approaches to the ADNI Plasma Proteome Dataset to identify protein signatures that might be useful for the diagnosis of pre-clinical AD.

We first consider the ability of univariate and multivariate plasma biomarkers to distinguish between cognitively normal controls and individuals with MCI who subsequently progress to AD. We demonstrate that the strongest single blood biomarker candidate on the ADNI panel, apolipoprotein E (APOE), is influenced by genotype independent of clinical diagnosis. We also identify an 11-analyte signature that accurately classifies participants into the correct clinical group and show that prediction accuracy is influenced by whether or not plasma APOE levels are taken into consideration.

We next assess the utility of biomarker signatures comprising ‘meta-features’ - functions involving two or more variables (e.g. the relative difference in abundance of two proteins) that have recently been shown by our group to enhance prediction accuracy in the AD plasma protein dataset of Ray and colleagues [13]. We demonstrate that consideration of meta-features can further improve classification accuracy, yielding signatures with >85% sensitivity and specificity.

We also assess the ability of plasma protein signatures to differentiate controls from patients with diagnosed AD, as well as to differentiate individuals with MCI who subsequently progress to AD from those who do not. Finally, we test our proposed signatures on data collected at a later time point, which highlights some of the limitations of using cross-sectional data to find biomarkers of pre-clinical AD, before going on to demonstrate how the use of longitudinal data might inform better biomarker selection.

## Materials and Methods

### ADNI Study

Data used in the preparation of this article were obtained from the ADNI database (adni.loni.ucla.edu). Details about the ADNI are given in the Acknowledgments section. Written informed consent was obtained from all participants and the study was conducted with prior institutional ethics approval. Further information about ADNI can be obtained from www.adni-info.org.

### ADNI Dataset

The methods used in the ADNI Plasma Proteome study are described in the document ‘Biomarkers Consortium ADNI Plasma Targeted Proteomics Project – Data Primer’ (available at http://adni.loni.ucla.edu).

Briefly, participants received a diagnosis at baseline of cognitively normal, MCI or AD based on clinical and neuropsychological testing. Participants were re-classified upon follow-up visits where appropriate. Plasma samples at baseline were collected from 58 cognitively normal controls, 396 individuals with MCI and 112 AD patients, selected based upon availability of additional biomarker endpoints (e.g. CSF Aβ_42_ levels or Pittsburgh Compound B imaging data). Plasma samples were also collected from a subset of these participants 12 months after baseline assessment. Plasma samples were assayed for 190 analytes using the ‘Human DiscoveryMAP’, developed on the Luminex xMAP platform by Rules-Based Medicine. Of the 58 individuals classed as cognitively normal at baseline, four have since been re-classified as either MCI or AD and were therefore excluded from analysis.

### Data Pre-Processing

Three levels of quality control were conducted for each analyte and are described in detail in the Data Primer. Analytes which were below assay detection limits in more than 10% of samples were excluded by the ADNI Analysis Team (*n* = 44). We conducted an assessment of raw data for each of the individual analytes that fit this criterion to ensure that undetectable samples were not disproportionately distributed across the different disease categories.

The ADNI dataset contains a ‘Least Detectable Dose’ (LDD) value for each analyte. This represents the concentration of analyte that produces a signal above the background level with 99% confidence (calculated from the average and standard deviation of readings of at least 20 ‘blank’ replicates) and is considered by ADNI as the most reliable lowest point for these protein assays. In these analyses, all readings for a given analyte below ½ LDD were converted to ½ LDD.

Where concentrations for analytes were not normally distributed, *log_10_* transformation was applied to facilitate summary statistics (this was required for all but nine analytes). For the initial analyses we have considered both raw data and *log_10_*-transformed data.

### Data Grouping

Unless otherwise stated, analyses described here were conducted on data generated from plasma samples collected at baseline. Data generated from plasma samples collected 12 months after baseline assessment were only used for validation studies and assessing change in analyte levels over time. Participant samples were separated into four groups: cognitively normal individuals (Control), participants with MCI at baseline that have since progressed to a diagnosis of AD (MCI Progressor), participants with MCI at baseline that have not yet progressed to a diagnosis of AD (MCI Other) and participants with AD at baseline (AD).

### Generation of Signatures

Analyte signatures were generated based on the *(α,β)-k-Feature Set problem* approach. This is a method based on techniques from combinatorial optimization and mathematical programming. It differs from statistical methods traditionally used in that it retains information about individual participants within each class rather than just considering univariate measures of class central tendency and variance. It also has the advantage of being a multivariate method that evaluates possible solutions involving sets of analytes, thereby maintaining the context of interrelationships between different analytes, which is often lost when considering each analyte individually. Different approaches based on the *(α,β)-k-Feature Set problem*, first introduced in 2004 [14], have been used to generate molecular signatures of various diseases, notably AD and prostate cancer [10], [13], [15], [16].

To generate signatures for a two-class comparison (e.g. Control vs. MCI Progressor) using this approach, the data are first preprocessed by filtering and discretization of the values. (An example is given below under ‘Classification – Univariate Markers’.) An implementation of Fayyad and Irani's algorithm [17], an entropy-based heuristic, is used to discretize data. This approach identifies, for each analyte, the concentration threshold with minimal class information entropy (a measure of how well a threshold separates the two classes) and discretizes the data based on this threshold. It then discards analytes that do not provide sufficient information to discriminate between the two classes under consideration, based on the Minimum Description Length principle (reviewed in [18]). This results in a dataset of binary values (representing analyte concentrations above or below the concentration threshold). By using binary data, the analysis results are not skewed by outlying values, as can occur with standard statistical approaches that compare group means.

The matrix of discrete values returned after entropy filtering is used to create an instance of the *(α,β)-k-Feature Set problem*. This models the problem of identifying a set of features (i.e. analytes, now discretized) that have maximum internal consistency in both distinguishing different sample classes (e.g. ‘health states’) and showing similarity within each sample class [18].

For each pair-wise grouping of samples in the dataset, the *(α,β)-k-Feature Set* problem considers the capacity of each feature (e.g. analyte) to describe the class labels (e.g. Control, MCI Progressor) of the two samples comprising this pair, based on the discrete values of a given feature for the samples in question. For example, when considering a pair comprising samples from different classes (e.g. one Control participant and one MCI Progressor participant), a given feature would be able to describe the class labels if its discrete values differed between the two samples. On the other hand, when considering a pair comprising samples from the same class (e.g. two Control participants), a given feature would be able to describe the class labels if its discrete values were the same in both samples.

The *(α,β)-k-Feature Set problem* involves three parameters; *α*, defined as the minimum number of features that must discriminate between any pair of samples from different classes; *β*, defined as the minimum number of features that must have the same discrete value for any pair of samples from the same class; and *k*, defined as the number of features (analytes) in the solution. In the present study, we have defined the optimal solution as one that achieves the minimal value for *k* with *α* = 1 and *β* = 1. In the case that more than one solution satisfies these conditions, we select the solution that provides the greatest ‘coverage’ of sample pairs belonging to different classes (we refer to [18] for details of this method). This approach has been applied previously by our group for feature selection in another AD plasma protein dataset [10], [13] and in a study of hippocampal gene expression in AD [15], as well as investigations of other diseases [16].

### Heat maps

Heat maps were generated by ordering analytes and samples using a high performance *Memetic Algorithm*. This technique is described elsewhere [18], [19] but briefly, it aims to minimize an objective function, in this case the correlation distance (where correlation distance = 1−correlation coefficient), both between different analytes and between different samples. This is achieved by applying an evolutionary approach, thereby improving upon the single-pass hierarchical clustering techniques that have been traditionally used for ordering large datasets [18], [19]. The solution achieved by the *Memetic Algorithm* is presented as an ordered matrix of rectangles colored along a green-red continuum (‘heat map’), with green representing lower expression values and red representing higher expression values.

### Classification

By determining which individual analytes pass the entropy filter and which set of analytes provide an optimal solution to the *(α,β)-k-Feature Set problem*, we can identify potential biomarkers that discriminate between two health states. However this process does not in itself directly provide a rule or formula that specifies in which health state an individual having particular values for the selected analytes should be classified. Instead, the potential utility of individual analytes or sets of analytes for classifying individuals into the correct health state was assessed as follows.

#### Univariate Markers

Analytes that passed the entropy filter (described above) were ranked by their ability, based on the binary values assigned in the discretization preprocessing step, to classify samples into the correct class (e.g. Control or MCI Progressor). As a hypothetical example, when comparing controls and MCI progressors, assume that for ‘analyte X’ the concentration threshold selected based on class information entropy is 50. All samples with an ‘analyte X’ concentration below this threshold are assigned a value of 0 and all samples above this threshold assigned a value of 1. We determine the percentage of control samples assigned a value of 0 for ‘analyte X’ and the percentage of MCI progressor samples assigned a value of 1, and vice versa. This process is repeated for all analytes that passed the entropy filter.

#### Multivariate Signatures

Classification of participants into one of two classes or ‘health states’ (e.g. Control or MCI Progressor) based on plasma levels of multiple analytes was performed using a number of different classification algorithms available in the Waikato Environment for Knowledge Analysis (WEKA) package [20]. The WEKA package contains 70 classifiers derived from different machine learning approaches (e.g. Bayes-based, tree-based, rules-based etc). We assessed the performance of each of the classifiers within the WEKA package across nine different signatures and selected 10 that consistently gave the highest Matthews Correlation Coefficient (see below) using a cross-validation approach (Table S1). These 10 classifiers subsequently used to test the ability of the different signatures to correctly classify participants into one of two health states. Results presented in this paper are the average of the data from the 10 different classifiers. (We note that after the analytes that comprise a particular signature have been identified, the discretized values are no longer used and instead we use the original values for the creation of classifiers.)

**Figure 1:**
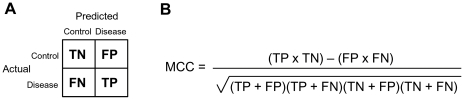
Calculation of Matthews Correlation Coefficient (MCC). (A) Contingency matrix illustrating our usage of true negatives (TN), false positives (FP), false negatives (FN) and true positives (TP). (B) Mathematical definition of the MCC.

For classifications in which only one dataset was used, a 10-fold cross-validation was conducted. This widely used approach involves randomly dividing the sample set into 10 subsets. The classifier then uses nine of these subsets as training data and one as a test set. This process is repeated 10 times, so that each of the 10 subsets is used exactly once as the test set. The results are then combined to give a single measure of sensitivity (proportion of affected participants correctly assigned to the disease class – true positives) and specificity (proportion of non-affected participants correctly assigned to the control class – true negatives) for the given signature (Figure 1a). As the size of the control group was substantially smaller than that of the MCI or AD groups when considering all samples, we also calculated the Matthews correlation coefficient (MCC; Figure 1b). This approach is recommended for binary data when class numbers are unequal and is preferred to simpler approaches, such as the average of the sensitivity and specificity, as it preserves information about all four components of the contingency matrix (Figure 1a) in an unbiased way [21].

There are presently no other publicly available datasets that are suitable for testing the signatures we have developed here. Instead, we also assessed the classification accuracy of the signatures using an artificial training/test set approach, in which samples from each health state were divided equally into one training set and one test set. The training and test set were matched for age, gender and *APOE* genotype but otherwise randomly assigned. Due to the disproportionate sizes of the different classes, we created single additional size-matched groups (e.g. 54 controls and 54 MCI progressors) that were age- and gender-matched but otherwise randomly assigned. For the small number of analyses where size-matched training and test sets were required, these groups were further sub-divided in one training set and one test set (e.g. *n* = 27 controls, 27 MCI progressors per set) that were also age- and gender-matched but otherwise randomly assigned.

Additionally, we assessed some of our proposed signatures on ADNI data from plasma samples collected 12 months after baseline assessment. These samples were collected from a subset of participants from the larger baseline group described above. This subset consisted of 50 controls, 92 MCI progressors and 97 AD patients.

## Results

### Demographics

The demographics of the sample at baseline are given in Table 1. (The demographics of the subset of participants for whom 12 month data were collected mirrored those of the larger cohort.) Age did not vary significantly between the different groups (*p-value* = 0.96, one-way ANOVA). The MCI and AD groups had a higher proportion of males to females than controls. Frequency of the *APOE*-ε4 allele, the main genetic risk factor for late-onset Alzheimer's disease [22], [23], was substantially higher in MCI and AD patients than controls. Mini-Mental State Examination (MMSE) score differed significantly between all groups (*p*<0.01), with Control>MCI Other>MCI Progressor>AD.

**Table 1:** Baseline demographics.

	Control	MCI		AD
		Other	Progressor	
**N Plasma samples**	54	233	163	112
**Mean age (range)**	75.4 (62–90)	75.0 (55–90)	74.8 (55–89)	75.4 (55–89)
**Gender M/F**	27/27	157/76	99/64	65/47
***APOE*** **-ε4 frequency**	9%	43%	67%	68%
**Mean MMSE score (range)**	28.9 (25–30)	27.3 (24–30)	26.6 (23–30)	23.6 (20–27)

### Comparison with proteomics dataset of Ray and colleagues

A highly-cited study by Ray and colleagues [9] generated a dataset of relative plasma protein levels in cognitively normal controls, individuals with MCI and AD patients and reported an 18-protein biomarker signature for distinguishing controls from AD patients. We have previously applied our analytical methods to the dataset contributed by Ray and colleagues [9], refining their 18-protein biomarker signature to a 5-protein signature [10] as well as identifying sets of protein pairs (i.e. the difference in relative abundance of two proteins) that can also accurately classify these participants [13].

**Figure 2:**
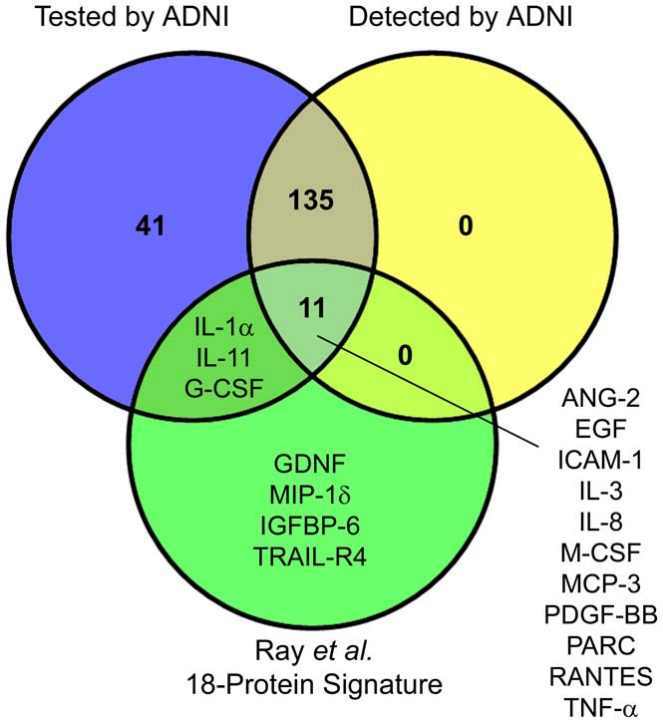
Detectability in the ADNI dataset of the 18 proteins highlighted by Ray *et al.* (2007). Of the 18 proteins in the signature highlighted by Ray and colleagues [9], three were below the detection limits of the ADNI assay, 11 were considered detectable by ADNI and four were not assessed. Protein abbreviations are defined in Table S2.

However the study of Ray and colleagues did not provide data on the absolute plasma levels of the different proteins assessed. We checked the proteins highlighted by Ray and colleagues in the ADNI dataset to gauge their level in plasma. Of the 18 proteins in the signature of Ray and colleagues [9], four were not included in the ADNI assay and three of the remaining 14 assessed by ADNI were below the detection limit of the Luminex assay (Figure 2, Table S2) – interleukin-1α, interleukin-11 and granulocyte colony stimulating factor. (Two of the five proteins in our reported signature based on the Ray dataset [10], as well as two of six proteins highlighted in our analysis of protein pairs from the Ray dataset [13], were also below the detection limit of the ADNI assay.)

### Control vs. MCI Progressor

One of the main questions driving this study was whether there exists a signature of plasma analytes that can successfully predict AD progression in pre-clinical participants and also distinguish these individuals from controls who do not progress to cognitive impairment (at least in the short term). For this reason we focused our attention on comparing baseline plasma analyte levels in cognitively normal controls who do not proceed to cognitive impairment (*n* = 54) with participants classified as MCI at baseline who have since been reclassified as AD (‘MCI Progressor’; *n* = 163).

#### Univariate analysis

Univariate statistical analysis identified APOE as the analyte differing most significantly between controls and MCI progressors, with lower levels in plasma from MCI progressors than controls. In statistical comparisons of the two groups (two-tailed *t-test*, adjusted using Welch's correction where appropriate), APOE returned the lowest *p* value (2.3×10^−13^) of all analytes assessed (Table S3). Only two analytes from the 18-protein signature reported by Ray and colleagues (Figure 2) showed statistically significant differences in the ADNI dataset - angiopoietin-2 and pulmonary and activation regulated chemokine.

We next applied a filtering step based on class information entropy (details in Methods) to eliminate analytes that did not provide good discrimination of control and MCI progressor samples. Of the 146 analytes considered detectable, 17 passed the entropy filter. These analytes were ranked by their ability, based on discrete values assigned by the entropy filter, to classify control and MCI progressor samples correctly (as described in Methods). Using this metric, APOE was again the analyte whose levels best discriminated the two groups based on MCC (Table 2). None of the analytes in the 18-protein signature reported by Ray and colleagues (Figure 2) passed the entropy filter.

**Table 2:** Accuracy of the analytes that passed entropy filtering in classifying control and MCI progressor samples.

Analyte	% Controls Correct (number)	% MCI Progressor Correct (number)	MCC
Apolipoprotein E	79.6 (43)	74.8 (122)	0.484
Apolipoprotein A-II	88.9 (48)	55.2 (90)	0.384
Macrophage Inflammatory Protein-1α	35.2 (19)	93.9 (153)	0.369
Eotaxin-3	16.7 (9)	100.0 (163)	0.361
Transthyretin	59.3 (32)	78.5 (128)	0.354
Brain Natriuretic Peptide	48.1 (26)	85.3 (139)	0.343
Heparin-Binding EGF-Like Growth Factor	64.8 (35)	71.2 (116)	0.321
α2-Macroglobulin	35.2 (19)	91.4 (149)	0.320
Calcitonin	48.1 (26)	82.8 (135)	0.310
Peptide YY	22.2 (12)	96.9 (158)	0.308
Betacellulin	72.2 (39)	63.2 (103)	0.307
Serotransferrin	53.7 (29)	77.9 (127)	0.298
C-Reactive Protein	55.6 (30)	76.1 (124)	0.294
Serum Glutamic Oxaloacetic Transaminase	85.2 (46)	47.2 (77)	0.287
Angiotensinogen	87.0 (47)	43.6 (71)	0.276
Fas Ligand	87.0 (47)	41.7 (68)	0.261
CD5	100.0 (54)	19.6 (32)	0.239

Analytes ordered by Matthews correlation coefficient (MCC). Control *n* = 54, MCI Progressor *n* = 163.

#### Effect of APOE genotype on APOE plasma levels

While these analyses suggest that plasma levels of APOE are a good marker of pre-clinical AD, it is possible that these results may be influenced by effects of *APOE* genotype, which modifies AD risk [22]–[26] and may also influence cognitive performance in non-demented individuals [27]–[30]. We therefore investigated whether *APOE* genotype can influence plasma APOE levels independent of clinical diagnosis.

**Figure 3:**
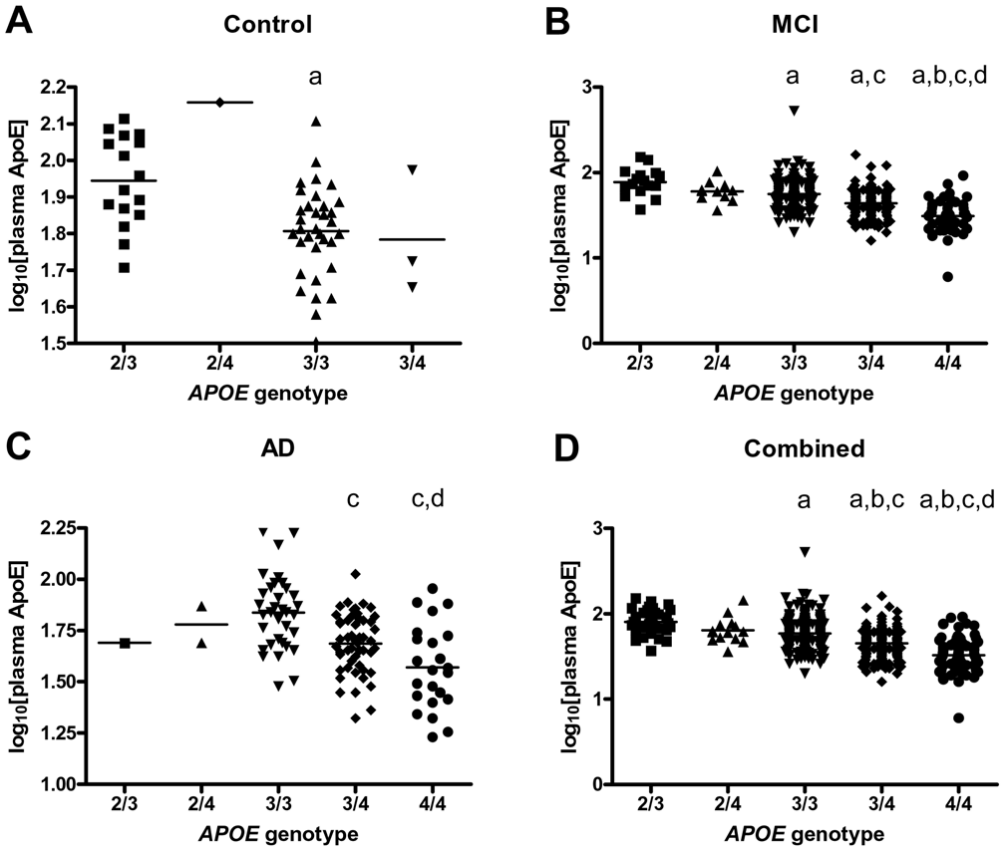
APOE plasma concentration as a function of *APOE* genotype. APOE concentration shows a decreasing trend from ‘protective’ (ε2) to ‘at risk’ (ε4) genotypes that is independent of clinical diagnosis. Plots illustrate APOE log_10_ plasma concentrations as a function of *APOE* genotype when considering samples classified at baseline as (A) control (*n* = 54), (B) MCI (*n* = 396) and (C) AD (*n* = 112). Plot (D) illustrates the trend when baseline diagnosis is not considered. 2/3 – *APOE*-ε2/ε3; 2/4 – *APOE*-ε2/ε4; 3/3 - *APOE*-ε3/ε3; 3/4 - *APOE*-ε3/ε4; 4/4 - *APOE*-ε4/ε4. Statistically significant (*p*<0.05) difference when compared to ^a^2/3, ^b^2/4, ^c^3/3, ^d^3/4. Statistical tests were not conducted on sample sets of *n*<4.

Plasma levels of APOE differed significantly with *APOE* genotype independent of clinical diagnosis (as assessed by one-way ANOVA with Tukey's multiple comparison test), with a progressive decrease in plasma APOE from ‘protective’ genotypes (ε2) to ‘at risk’ genotypes (ε4; Figure 3). In view of this finding, all subsequent analyses were conducted on two sets of data - one that included the plasma APOE analyte and one that excluded the APOE analyte. (This is not to be confused with analyses that exclude participants with a particular *APOE* genotype.)

#### Multivariate analysis

We next applied a multivariate method, based on finding the smallest solution to the *(α, β)-k-Feature Set problem* (Methods), to identify a set of analytes (signature) that together best discriminate control samples from MCI progressor samples. The signature that has the smallest number of analytes and is a solution to the feature set problem (for *α* = 1 and *β* = 1 and maximum coverage) contains 11 analytes (Table 3, Figure S1a). No solution was possible when the APOE analyte was excluded from consideration (i.e. not every sample pair could be described or ‘covered’ by at least one analyte). Nonetheless, to determine whether a signature without APOE can still be useful for classifying most participants, we generated the optimal set (the highest possible values for *α* and *β* and greatest ‘coverage’) when constraining the size of the signature to 11 analytes (Table 3, Figure S1b). Identical signatures are obtained for both *log_10_*-transformed data and raw data.

**Table 3:** Feature set selection of 11-analyte signatures to discriminate control and MCI progressor samples.

Analyte (abbreviation)	
a) Including APOE	b) Excluding APOE
* *α2-Macroglobulin (α2M)*	* *α2-Macroglobulin*
*Angiotensinogen*	*Angiotensinogen*
*Apolipoprotein A-II (ApoA-II)*	*Apolipoprotein A-II*
Apolipoprotein E (ApoE)	Brain Natriuretic Peptide (BNP)
*Betacellulin (BTC)	CD5
Fas Ligand (FasL)	Eotaxin-3
*Heparin-Binding EGF-Like Growth Factor (HB-EGF)*	*Heparin-Binding EGF-Like Growth Factor*
*Macrophage Inflammatory Protein-1α (MIP-1α)*	*Macrophage Inflammatory Protein-1α*
*Peptide YY (PYY)*	*Peptide YY*
*Serum Glutamic Oxaloacetic Transaminase (SGOT)	*Serotransferrin (Tf)
*Transthyretin (TTR)*	*Transthyretin*

Signatures were generated using baseline data on 54 Control and 163 MCI Progressor participants. Analytes in italics were selected in both signatures (i.e. independent of the inclusion/exclusion of APOE).

* Analytes that passed the entropy filter and were selected in the signature but did not show statistically significant (*p*<0.01) differences between controls and MCI progressors (Table S3).

The ability of these signatures to correctly classify the full set of controls and MCI progressors was assessed with the WEKA package using both a 10-fold cross-validation approach and an artificial training/test set approach (Methods). Using each of 10 different classifiers based on various different machine learning models (Table S1), the signature containing the APOE analyte achieved an average sensitivity of over 90% but a specificity of less that 70%. The signature excluding the APOE analyte had a comparable sensitivity but an even lower specificity (Table 4).

**Table 4:** Accuracy of analyte signatures in classifying controls and MCI progressors.

Signature	Cross-Validation			Training Set			Test Set		
	Sens	Spec	MCC	Sens	Spec	MCC	Sens	Spec	MCC
Signature with APOE									
a *Full set of samples*	93.5	66.9	**0.64**	97.8	93.7	0.92	93.0	64.8	**0.61**
b *Size-matched groups*	74.3	79.3	**0.54**	97.8	93.7	0.92	85.9	64.8	**0.52**
Signature without APOE									
*Full set of samples*	92.0	54.3	0.50	97.7	85.6	0.86	91.6	46.3	0.43
*Size-matched groups*	67.2	73.5	0.41	91.1	96.3	0.88	77.0	71.1	0.50
Top 11 analytes - *p* val									
*Full set of samples*	91.3	60.0	0.54	96.8	90.7	0.88	91.5	56.3	0.51
*Size-matched groups*	70.2	70.6	0.41	90.7	94.8	0.86	84.4	64.4	0.51
All 146 analytes									
*Full set of samples*	94.0	57.4	0.57	99.4	97.8	**0.97**	95.1	53.3	0.55
*Size-matched groups*	69.1	73.5	0.43	98.9	99.3	**0.98**	71.5	70.7	0.42

Classification accuracy was tested using *log_10_*-transformed data. We have used bold font to draw particular attention to the signatures with the best performance (as assessed by MCC) in each comparison (i.e. 10-fold cross-validation, training set or test set). As expected, using all 146 analytes leads to some overfitting on the training set but our signatures that include the APOE analyte perform better in test scenarios.

a The full set of samples contained data on 54 Controls and 163 MCI Progressors.

b The size-matched groups contained data on 54 Controls and 54 MCI Progressors. For each of these datasets, all samples were used for cross-validation, whereas training and test sets were created by dividing datasets into two equal subsets.

As the use of raw data resulted in identical signatures and comparable classification accuracy to *log_10_*-transformed data, all further analyses were conducted on *log_10_*-transformed data only, which have a normal distribution and could therefore be used for Z-score transformation for heat maps and consideration of analyte pairs (below).

#### Influence of group size on measures of sensitivity and specificity

As described above, when tested within the various classifiers on the unequal Control and MCI Progressor groups (where there were three times as many MCI progressors as controls), the multivariate signatures returned low values for specificity (i.e. accuracy in correctly classifying controls) relative to sensitivity (i.e. accuracy in correctly classifying MCI progressors). This would be problematic in a clinical setting, where it is important not to misdiagnose healthy individuals as having an incurable, terminal condition.

We hypothesized that the discrepancy observed between values for sensitivity and specificity (irrespective of whichever classifier was used) was an artefact of having disproportionately sized groups, as classification algorithms in general apply strategies that optimize total accuracy (i.e. in this case, the total number of correctly called controls and MCI progressors). This biases correct classification towards groups with larger sample size, as is the case here for MCI progressors. To determine the effect of group size on tuning the classification strategies and subsequent values for sensitivity and specificity, we tested our signatures on size-matched groups of MCI progressors and controls (*n* = 54 per group). In most tests, matching of group sizes gave sensitivity and specificity results that were less discrepant than when considering the full set of samples. Across the different classification approaches, sensitivity and specificity were generally in the range of 65–85% (Table 4).

To evaluate the effectiveness of our feature selection method relative to selection strategies based on statistical measures, we assessed the classification accuracy of a signature comprising the 11 analytes showing the most statistically significant differences between controls and MCI progressors. Our signature out-performed the statistics-based signature in all classification tests performed (Table 4). We also assessed whether using the full set of available plasma analyte data provided more accurate classification than our 11-analyte signature. As expected, using data on all 146 analytes gave highly accurate classification on a training set, however it resulted in a poorer performance (based on MCC) when assessed by cross-validation or applied to test sets (Table 4), probably due to overfitting of the data.

We also generated new signatures using the size-matched dataset of 54 controls and 54 MCI progressors. A total of 11 analytes passed the entropy filter (Table S4), all of which were identified when considering the full set of samples with the exception of Tamm-Horsfall Urinary Glycoprotein. Classification of control and MCI progressor samples within the size-matched dataset gave a sensitivity and specificity comparable to those obtained when assessing the original signatures (Table S5).

#### Influence of demographics and genotype on measures of sensitivity and specificity

Age and gender are known to influence the levels of particular blood proteins and, while the Control and MCI Progressor groups were matched for both age and gender, it is possible that the accuracy of the proposed signatures in correctly classifying individuals from these two groups may vary depending on age and gender. To investigate this possibility, we stratified the size-matched groups into subsets containing exclusively male or female participants (*n* = 27 for each gender) and subsets stratified as ‘younger’ or ‘older’ participants (based on the median age of each group). Classification using a 10-fold cross-validation approach showed no discernible difference between measures of sensitivity and specificity when signatures were applied to male and female groups separately. In comparisons of the ‘younger’ and ‘older’ subsets, the signature containing the APOE analyte performed slightly better in classifying participants in the ‘older’ subset i.e. above the median age (Table S6), however neither signature performed better on age-stratified subsets than on the larger non-stratified group.

Because of the potential confounding effect of *APOE* genotype, we also considered a restricted sample that included only individuals with homozygous *APOE*-ε3 genotypes (34 controls, 50 MCI progressors). In this restricted group, APOE no longer passed the entropy filter of analytes discriminating between controls and MCI progressors (Table S7, Figure S2). Of the six analytes passing the entropy filter, all but one (α1-antitrypsin) were previously identified when considering the full set of samples, suggesting that these markers are robust against the influence of *APOE* genotype. This set of six analytes was able to classify *APOE*-ε3 homozygote controls and MCI progressors with an average sensitivity of 83.6% and a specificity of 77.6% (MCC = 0.61).

Finally, as age, gender and *APOE* genotype are all known to influence risk of AD, we assessed the ability of these three attributes, as a collective, to discriminate controls from MCI progressors. Using a ‘signature’ comprising these three variables, classification of a size-matched dataset of Control and MCI Progressor participants achieved a sensitivity of 71% and a specificity of 80%. This was largely driven by *APOE* genotype (which alone gives an accuracy of almost 80%) rather than age or gender.

### Meta-feature analysis

Our group has previously demonstrated that incorporating ‘meta-features’ into biomarker signatures can improve classification accuracy [13]. A meta-feature can be considered as any function that involves more than one feature, such as the difference in abundance of two analytes. Here and in the previous study [13], for simplicity, we only consider meta-features based on two variables and a simple arithmetic function. This is a generalization of a common practice in classification. For instance, consideration of the ratio of concentrations of two different analytes has already been used to successfully establish CSF biomarkers of AD from univariate analysis (e.g. Aβ_42_/tau) [31], [32]. Using meta-features based on concentration ratios for biomarker discovery can help mitigate any confounding effects due to inter-sample biological variability or technical variability (e.g. differences in the volume of sample assayed), as the two analytes are jointly measured and their concentrations relative to one another determined within each individual sample. The use of meta-features can also facilitate the identification of features that are mathematically or biologically dependent in a supra-additive (synergistic) way with regard to disease prediction capacity i.e. potentially inter-related (whether directly or not). We therefore calculated differences and sums of Z-score values for each possible analyte pair, which is equivalent to calculating the ratio and product of the relative abundance of two analytes.

#### Pair-wise differences

When considering differences in Z-scores of analyte pairs for controls against MCI progressors, 1,141 meta-features passed the entropy filter from a total of 10,585. As described above for single analyte analysis, each meta-feature passing the entropy filter was assessed for its ability, based on discrete values assigned by the entropy filter, to classify control and MCI progressor samples correctly. Meta-features were then ordered based on this metric and the top 100 ranked meta-features were used to generate a signature. (Generating a signature from the full set of meta-features passing the entropy filter was not computationally feasible.) When APOE-related meta-features were included, the smallest signature that produced a solution to the feature set problem (with *α* = 1 and *β* = 1 and maximum coverage) contained 8 meta-features (3 of which involved APOE) and involved 13 different analytes. When APOE was excluded from consideration, the smallest signature that produced a solution contained 7 meta-features, which also involved 13 different analytes (Table 5, Figure S3). The smaller size of the signature when APOE-related meta-features were excluded is probably due to the dominance of APOE among the top 100 ranked meta-features passing the entropy filter. When the APOE-related meta-features were removed from consideration, a large number of new meta-features were introduced into the top 100 list – these in turn contributed to a new solution that required less meta-features.

**Table 5:** Minimal meta-feature set selection of differences in analyte abundances to discriminate Control and MCI Progressor samples.

Analyte pairs (abbreviation)	
a) Including APOE	b) Excluding APOE
Angiopoietin-2 & Interleukin-16 (ANG-2 – IL-16)	*α1-Microglobulin & Apolipoprotein A-II (α1M – ApoA-II)*
*Apolipoprotein A-II & Betacellulin (ApoA-II – BTC)*	Angiopoietin-2 & Neuronal Cell Adhesion Molecule (ANG-2 – Nr-CAM)
*Apolipoprotein E & Brain Natriuretic Peptide (ApoE – BNP)*	*Angiopoietin-2 & Transthyretin (ANG-2 – TTR)*
*Apolipoprotein E & Serotransferrin (ApoE – Tf)*	Apolipoprotein D & Insulin-like Growth Factor-Binding Protein 2 (ApoD – IGFBP-2)
*Apolipoprotein E & Thrombopoietin (ApoE – Thrombopoietin)*	C-Reactive Protein & Pregnancy-Associated Plasma Protein A (CRP – PAPP-A)
*Chromogranin-A & Heparin-Binding EGF-Like Growth Factor (CgA – HB-EGF)*	*Macrophage Inflammatory Protein-1α & Pulmonary and Activation-Regulated Chemokine (MIP-1α – PARC)*
*Interleukin-6 receptor & Macrophage Inflammatory Protein-1α (IL-6r – MIP-1α)*	Matrix Metalloproteinase-10 & Matrix Metalloproteinase-9 (MMP-10 – MMP-9)
*Macrophage Inflammatory Protein-1α & Pulmonary and Activation-Regulated Chemokine (MIP-1α – PARC)*	

Signatures were generated using baseline data on 54 Control and 163 MCI Progressor participants. Meta-features in italics contain at least one analyte identified in the corresponding signature generated by single analyte analysis (Table 3).

These meta-feature signatures achieved higher classification accuracy than the signatures involving single analytes described above. In addition, the exclusion of APOE-related meta-features from the signature did not profoundly affect prediction accuracy. When considering the size-matched groups of controls and MCI progressors, the signatures containing pair-wise differences achieved a sensitivity and specificity of greater than 85%, irrespective of the classification approach used (Table 6).

**Table 6:** Accuracy of meta-feature signatures involving differences in analyte abundances in classifying controls and MCI progressors.

Signature	Cross-Validation			Training Set			Test Set		
	Sens	Spec	MCC	Sens	Spec	MCC	Sens	Spec	MCC
8-metafeature signature with APOE									
a *Full set of samples*	94.7	78.1	0.74	99.1	96.3	0.96	93.2	64.8	0.61
b *Size-matched groups*	90.2	87.2	0.77	98.9	98.1	0.97	85.6	86.7	0.73
7-metafeature signature without APOE									
*Full set of samples*	95.6	78.1	0.76	97.9	95.9	0.93	96.0	72.6	0.73
*Size-matched groups*	83.3	87.6	0.71	90.7	99.2	0.91	90.7	83.0	0.74

a The full set of samples contained data on 54 Controls and 163 MCI Progressors.

b The size-matched groups contained data on 54 Controls and 54 MCI Progressors. For each of these datasets, all samples were used for cross-validation, whereas training and test sets were created by dividing datasets into two equal subsets.

To ensure that the improved performance of meta-feature signatures was not solely the result of using 13 different analytes rather than 11 (the number comprising single analyte signatures above), we used the *(α,β)-k-Feature Set problem* approach to determine the optimal solution containing 13 single analytes, as well as creating a signature comprising the 13 analytes showing the most statistically significant differences between controls and MCI progressors. In all classification tests conducted, the meta-feature signatures correctly classified a higher proportion of both controls and MCI progressors than the single analyte signatures (Table S8), suggesting that consideration of meta-features can be a valuable tool to supplement more traditional approaches to biomarker discovery.

#### Pair-wise sums

We also considered the utility of meta-features involving pair-wise sums. A total of 980 meta-features passed the entropy filter, of which 8 were required to produce a solution to the feature set problem (Table S9, Figure S4). Unlike the signatures comprising pair-wise differences discussed above, the consideration of pair-wise sums did not result in markedly improved classification accuracy when compared to signatures containing single analytes (Table S10).

### Summary of prediction accuracy of different signatures

In summary, the multivariate plasma analyte signatures identified here effectively discriminated a high proportion of MCI progressors from cognitively normal controls. When the signatures were tested on the full set of participants, biases in the classification algorithms led to the correct classification of a high proportion of MCI progressors but a relatively low proportion of controls. Tuning the classification algorithms on size-matched control and MCI progressor groups, although resulting in a reduction in MCC values, mostly eliminated the disparity between values for sensitivity and specificity that occurred when classifying groups of disproportionate sizes. Relative to signatures comprising sets of single analytes, consideration of pair-wise differences generally resulted in a further improvement in sensitivity and specificity, particularly for signatures in which APOE-related features were excluded, whereas consideration of pair-wise sums had little effect.

### Control vs. AD

Similar to comparisons of controls and MCI progressors above, univariate statistical analysis revealed APOE as the analyte differing most significantly between controls and AD patients, with lower levels in plasma from AD patients relative to controls. In statistical comparisons of the two groups (two-tailed *t-test*, adjusted using Welch's correction were appropriate), APOE returned the lowest *p-value* (5.2×10^−7^) of all analytes assessed (Table S11).

The multivariate signatures comprising single analytes that were generated when comparing control and MCI progressor groups were applied to data from AD samples to determine the accuracy of these signatures in classifying AD patients. The set of 10 classifiers were trained on sized-matched sets of controls and MCI progressors and then tested on the full set of AD patients (*n = 112*). The signature containing APOE accurately predicted AD in 67.0% of patients, while the signature excluding APOE accurately predicted 65.9%. Not surprisingly, when classifiers were trained on the full set of controls and MCI progressors (which biases classification towards higher sensitivity), the accuracy of predicting AD improved substantially. The signature including APOE accurately predicted AD in 79.2% of patients, while the signature excluding APOE accurately predicted 85.2%. As APOE showed such a strong difference between controls and AD patients, it may seem surprising that the signature with APOE correctly classified a lower percentage of AD patients than the signature without APOE. This may be due to the latter signature containing other analytes that better discriminate between control and AD than those included in the APOE-containing signature, for example eotaxin-3, which, as discussed below, best discriminated the control and AD groups based on MCC (Table 7).

**Table 7:** Accuracy of the analytes that passed entropy filtering in classifying control and AD samples.

Analyte	% Controls Correct (number)	% AD Correct (number)	MCC
Eotaxin-3	63.0 (34)	80.4 (90)	0.429
Brain Natriuretic Peptide	48.1 (26)	88.4 (99)	0.404
Serum Glutamic Oxaloacetic Transaminase	85.2 (46)	54.5 (61)	0.377
Apolipoprotein E	79.6 (43)	58.0 (65)	0.354
α1-Microglobulin	75.9 (41)	61.6 (69)	0.352
Betacellulin	83.3 (45)	53.6 (60)	0.351
Apolipoprotein A-II	88.9 (48)	46.4 (52)	0.347
Pregnancy-Associated Plasma Protein A	87.0 (47)	48.2 (54)	0.343
Peptide YY	85.2 (46)	50.0 (52)	0.339
Placenta Growth Factor	100 (54)	25.0 (28)	0.313
Receptor for Advanced Glycosylation End Product	90.7 (49)	39.3 (44)	0.308
CD5	100 (54)	20.5 (23)	0.278
Immunoglobulin M	100 (54)	15.2 (17)	0.235

Analytes ordered by Matthews correlation coefficient (MCC). Control *n* = 54, AD *n* = 112.

We next looked at whether a different set of analytes might better discriminate between controls and AD patients than the sets previously determined from comparisons of the control and MCI progressor groups. To assess this, we compared baseline plasma analyte levels in cognitively normal controls who did not proceed to cognitive impairment with those in patients classified as AD at baseline.

Of the 146 analytes considered detectable, 13 passed the entropy filter. As described in Methods, these analytes were ranked by their ability, based on discretized values assigned by the entropy filter, to classify AD and control samples correctly (Table 7). Using this metric, as mentioned above, eotaxin-3 best discriminated the two groups based on MCC. However the findings suggest that assessing eotaxin-3 alone would result in misclassifying a substantial proportion of healthy controls, which, as noted above, would be generally considered unacceptable in a clinical setting. Instead it might be preferable to trade sensitivity for specificity and select a marker that can correctly classify a higher percentage of controls, such as serum glutamic oxaloacetic transaminase.

The smallest signature that produced a solution (i.e. *α* = 1, *β* = 1) to the feature set problem contained 11 analytes or, when the APOE analyte was excluded, 12 analytes (Table S12, Figure S5). While these signatures showed some similarities to the signatures selected above for discriminating controls and MCI progressors (shaded in Table S12), there were several differences. These signatures achieved a sensitivity of around 85% but specificity of less than 65% when assessed on the full set of control and AD samples. When assessed on size-matched groups, sensitivity and specificity were more comparable but still relatively low (less than 75%; Table S13). These values are lower than those achieved when using multivariate signatures to classify controls and MCI progressors, possibly due to the lower number of samples in the AD group relative to the MCI Progressor group.

We also investigated whether the signatures generated by comparing controls and AD patients could effectively discriminate controls from MCI progressors. When tested on size-matched groups of controls and MCI progressors, the signature containing the APOE analyte returned a sensitivity of 67% and specificity of 76%, while the signature excluding APOE returned the same sensitivity but a lower specificity (66%). These values were considerably lower than those achieved by signatures derived from direct comparison of the Control and MCI Progressor groups (Table 4).

### MCI Progressor vs. MCI Other

In addition to biomarker signatures that can distinguish healthy controls from pre-clinical and clinical AD patients, it would be informative to be able to discriminate MCI patients who are likely to progress to AD from those who are not. We therefore attempted to generate a signature when comparing participants with MCI at baseline who have since progressed to AD (MCI Progressor; *n = 163*) and participants with MCI at baseline who have not yet progressed to AD (MCI Other; *n = 233*). Statistical comparisons of the two groups by *t-test* revealed 9 analytes that differed significantly in their levels (*p*<0.05, unadjusted for multiple testing; Table S14). Of these, macrophage inflammatory protein-3α had the lowest *p*-value (0.0015). However none of the analytes had sufficient discriminatory power to pass the entropy filter. As discussed further below, the lack of strong discriminators in this comparison may be due to heterogeneity within the MCI Other group, where some participants may progress to AD in the future, some may progress to different dementias and others may remain with MCI or even revert to control status.

To reduce heterogeneity within the MCI Other sample, we excluded participants who were re-classified as cognitively normal controls at one of the clinical follow-up visits (*n* = 19), however there were still no analytes that passed the entropy filter.

### Validation of signatures using data from 12 month follow-up

For a number of participants assessed at baseline, plasma analyte concentrations were also measured during a follow-up visit 12 months later. For clinical utility, ideally a signature should be robust both against technical variation and against biological variation which is unrelated to the condition of interest. To evaluate the broader applicability of the previously derived signatures, we assessed the classification accuracy of these signatures on plasma protein data obtained from participants at 12 months follow-up.

#### Control vs. MCI Progressor

For the participants used in the baseline analysis described above, 12 month data were available for 50 controls and 92 MCI progressors (all MCI progressors who had converted to AD in the period between baseline and 12 month evaluations were excluded from analysis). When the previous signatures were used to classify participants based on the data at 12 months, the sensitivity and specificity were low compared to the sensitivity and specificity achieved by these same signatures on the baseline data, for both the full set of participants and size-matched groups (Table 8).

**Table 8:** Validation of proposed signatures on data collected at 12 month follow-up.

Signature	Sens	Spec	MCC
**Control v MCI Progressor – Single Analyte Signature**			
Signature with APOE			
a *Full set of samples*	79.3	56.2	0.36
b *Size-matched groups*	65.8	70.8	0.37
Signature without APOE			
a *Full set of samples*	79.1	37.0	0.18
b *Size-matched groups*	57.4	49.6	0.07
**Control v MCI Progressor – Pair-Wise Differences Signature**			
Signature with APOE			
a *Full set of samples*	71.7	45.4	0.17
b *Size-matched groups*	58.2	59.8	0.18
Signature without APOE			
a *Full set of samples*	81.2	42.8	0.27
b *Size-matched groups*	66.2	59.4	0.26
**Control v AD – Single Analyte Signature**			
Signature with APOE			
c *Full set of samples*	81.9	68.6	0.51
d *Size-matched groups*	72.8	81.4	0.55
Signature without APOE			
c *Full set of samples*	79.4	55.6	0.37
d *Size-matched groups*	61.6	69.6	0.32

a The full set of samples contained data on 50 Controls and 92 MCI Progressors.

b The size-matched groups contained data on 50 Controls and 50 MCI Progressors.

c The full set of samples contained data on 50 Controls and 97 AD patients.

d The size-matched groups contained data on 50 Controls and 50 AD patients.

We next applied the meta-feature signatures involving the difference in relative levels of pairs of analytes to the 12 month data. As for the single analyte signatures, the meta-feature signatures were not effective in discriminating controls and MCI progressors in the 12 month data (Table 8).

#### Control vs. AD

In addition, we assessed the classification accuracy of the previously derived signatures for discriminating controls from AD patients using the 12 month data. Of the participants used in the baseline analysis described above, 12 month data were available for 50 controls and 97 AD patients (individuals who had converted to AD in the period between baseline and 12 month evaluations were not included in this analysis). These signatures performed better on 12 month data than the signatures for discriminating controls from MCI progressors discussed in the preceding paragraph, returning sensitivity and specificity values comparable to those achieved with baseline data (Table 8).

### Using the change in analyte concentration over time as a biomarker

We hypothesized that better accuracy in discriminating controls and MCI progressors might be achieved by assessing the change in analyte levels within individual participants over time. Longitudinal analyses of this kind essentially provide an internal control for non-disease-related variation by normalizing baseline values across individuals. This can mitigate the effect of factors such as age, gender and genotype, which may affect cross-sectional data.

To address this hypothesis, we calculated the change in analyte concentration from baseline to 12 months follow-up. For *log_10_*-transformed data, this was calculated by subtracting the baseline value from the 12 month value. For raw data, this was calculated by dividing the 12 month concentration by the baseline concentration. In order to eliminate from consideration analytes showing non-AD related variation, we excluded any analyte that changed on average more than 20% from baseline to 12 months in individuals from the Control group, leaving 110 analytes.

Data were then entropy filtered and feature set analysis performed as described above. Of the 110 analytes assessed, 26 passed the entropy filter. The smallest signature that produced a solution (i.e. *α* = 1, *β* = 1) to the feature set problem contained eight analytes (Table 9). (While the APOE analyte passed the entropy filter, it was not present in this signature and therefore did not need to be excluded from consideration as it was in previous analyses). In classifying controls and MCI progressors, the signature from this longitudinal analysis gave higher values of sensitivity and specificity than the corresponding signatures generated from cross-sectional data. When tested on the full set of samples (50 controls, 92 MCI progressors), the signature achieved a sensitivity of 88.2% and a specificity of 76.2% (MCC = 0.65). Testing on size-matched groups (50 controls, 50 MCI progressors) returned a sensitivity of 81.6% and a specificity of 83.8% (MCC = 0.66). However this signature did not effectively discriminate progressors from non-progressors within the MCI group (sensitivity = 57.6%, specificity = 48.0%).

**Table 9:** Feature set selection of signatures to discriminate control and MCI progressor samples based on longitudinal change.

a) Single Analyte Longitudinal Signature
Chemokine CC-4
Complement Factor H
Cystatin C
Interleukin-16
Kidney Injury Molecule 1
Macrophage Inflammatory Protein-1α
Resistin
Sortilin

b) Pair-Wise Differences Longitudinal Signature
Adiponectin & Complement Factor H
Cancer Antigen 19-9 & Macrophage Inflammatory Protein-1α
CD 40 antigen & Complement Factor H
Chemokine CC-4 & E-Selectin
Immunoglobulin A & Matrix Metalloproteinase-7

Signatures were generated using longitudinal data of the change in analyte levels over 12 months on 50 Control and 92 MCI Progressor participants. a) Signature generated when considering the change in individual analytes. b) Signature generated when considering the change in values for pair-wise difference metafeatures.

Similarly, we generated a signature based on the change in meta-feature values (pair-wise differences) over the 12 month period. After restricting the dataset to meta-features that did not show a substantial change from baseline to 12 months in individuals from the Control group, the smallest signature that produced a solution (i.e. *α* = 1, *β* = 1) contained five meta-features (Table 9). When tested on the full set of samples (50 controls, 92 MCI progressors), the signature achieved a sensitivity of 94.0% and a specificity of 87.4% (MCC = 0.82). Testing on size-matched groups (50 controls, 50 MCI progressors) returned a sensitivity of 89.2% and a specificity of 92.2 (MCC = 0.82), yet this still failed to improve discrimination within the MCI group (sensitivity = 53.7%, specificity = 55.4%).

In addition, we directly compared longitudinal data from the MCI Progressor and MCI Other groups using several approaches, including both combinatorial optimization and conventional statistics, but were still unable to identify either univariate or multivariate markers that discriminate progressors from non-progressors (data not shown).

In conclusion, we have identified signatures which are highly effective in discriminating controls from MCI progressors or AD patients but neither the cross-sectional nor longitudinal signatures were able to effectively discriminate progressors from non-progressors within the MCI group. This appears to relate to real biological heterogeneity within the MCI group and not limitations of any one particular feature selection method.

## Discussion

In this independent analysis of the ADNI Plasma Proteome dataset, we have demonstrated the value of a novel multivariate feature selection approach for identifying signatures of plasma analytes that distinguish pre-clinical AD from healthy controls more effectively than a collection of the ‘best’ markers as determined by statistical univariate analysis. The important difference between this type of approach and other more conventional analyses of putative blood biomarkers is that it considers information about individual participants rather than just assessing univariate measures of class central tendency and variance. As a result, a signature set will sometimes contain analytes that do not vary significantly between groups of control and test samples yet still contribute to distinguishing these groups through the contrast between their behaviour and that of other analytes within individuals. This is not taken into consideration by univariate approaches that assess the levels of single analytes in isolation. In addition, particular analytes that do not vary significantly between two large groups can sometimes provide information about a subset of samples with profiles that are not consistent with the majority of the sample pool. It is therefore important to stress that the unitary components of a multivariate signature should not be trialled as stand-alone univariate biomarkers but instead need to be validated in the context of all the analytes comprising that signature, using appropriate classification algorithms.

The analytes that were measured by the ADNI study were not all selected because of specific links with AD, however a number of the analytes comprising the signatures we identified have been shown previously to be altered in blood or CSF from people with MCI or AD (Table S15). Notably, other studies have found α2-macroglobulin levels to be higher in plasma from AD patients [33] and transthyretin and transferrin levels to be lower in serum from AD patients than controls [34], [35], consistent with the findings from the ADNI plasma dataset. While it is difficult to directly compare the longitudinal and meta-feature analyses with the single analyte comparisons previously reported in the literature, several of the longitudinal signature components have also been potentially implicated in AD through previous biomarker studies. These include cystatin C, sortilin and kidney injury molecule 1, which have been identified in an independent analysis of ADNI CSF samples [36], In addition, sortilin shows similarity to the sortilin-related receptor SORL1 that is genetically associated with AD risk [37], [38]. It is noteworthy that the directions of the longitudinal effects observed for these analytes in plasma appear consistent with the directions of changes in AD patients relative to controls in the ADNI CSF study discussed above.

It is striking that the majority of the analytes involved in the longitudinal signatures have been highlighted in the literature as important in renal disease in particular and, often in relation to this, in cardiovascular disease or diabetes. Notable examples include cystatin C, kidney injury molecule 1, cancer antigen 19-9, complement factor H and macrophage inflammatory protein 1α. However, while this suggests that differences relating to these conditions may exist between controls and MCI progressors, not all of the group differences were in directions that would normally be associated with pathogenicity and some may instead reflect compensatory mechanisms, complicating interpretation.

While there have been no published studies which have used the ADNI plasma dataset to identify signatures that distinguish controls from MCI progressors, one recently published study by O'Bryant and colleagues used the ADNI dataset to test the classification accuracy of a signature designed to discriminate cognitively normal controls from patients with clinical AD [39]. This signature, which was selected based on serum biomarker data from the Texas Alzheimer's Research Consortium, returned a sensitivity of 54% and specificity of 78% when tested on baseline ADNI data. The authors reported that as seen in other studies, accuracy was improved substantially by incorporating demographic and clinical lab data. Similar to our signatures for discriminating controls and AD patients, the signature of O'Bryant and colleagues comprised a total of 11 analytes, however only one of the analytes in this signature (tenascin C) passed the entropy filter used in our analyses. While we have not conducted a detailed analysis of how the multitude of clinical and demographic variables collected by ADNI can be combined with plasma protein data to generate signatures with improved classification accuracy, this is likely to be an important direction for future analyses.

We have previously applied our analysis approach to the plasma proteomic dataset contributed by Ray and colleagues [9], refining their 18-protein biomarker signature for distinguishing controls from AD patients to a 5-protein signature [10]. However, as described above, there are inconsistencies between the ADNI dataset and the dataset of Ray and colleagues. This discrepancy may reflect differences in the participant cohort, differences in the sensitivity of the assays used by the two studies, differences in the selection of thresholds to eliminate background noise or other factors. The Luminex technology used in the ADNI study has been well validated and the assay protocols include stringent quality controls, measurement of standards to allow calculation of the absolute concentration of plasma analytes and calculation of the least detectable dose (LDD) to facilitate identification of unreliably low readings. Further assessment of these different techniques is required to explain the discrepancies.

In addition, the study by Ray and colleagues did not assess *APOE* genotype. Our analyses show that *APOE* genotype has important effects on plasma levels of APOE and possibly other biomarkers. The finding that levels of APOE in the plasma are affected by *APOE* genotype is consistent with previous studies, which have demonstrated a gradient of APOE plasma concentrations as a function of *APOE* genotype (ε2>ε3>ε4) [40]–[43]. It is well established that plasma concentrations of total cholesterol and low density lipoprotein cholesterol also differ considerably depending on *APOE* genotype [44]–[48] and it is feasible that levels of various plasma proteins are regulated in response to changes in cholesterol or APOE levels. In support of this, *APOE* genotype has previously been associated with changes in blood levels of apolipoprotein A [49], apolipoprotein B [44], [45], [48], [49] and C-reactive protein [50], [51]. The effect of *APOE* genotype on the putative plasma biomarkers explored here highlights the importance of first considering an individual's *APOE* genotype if a plasma biomarker panel is to be used as a diagnostic tool. It may even be necessary to test different biomarker signatures depending on *APOE* genotype, particularly in view of the variability in frequencies of different *APOE* alleles across different populations [52], [53].

While the effect of *APOE* genotype on plasma APOE concentration observed here was independent of clinical diagnosis, it is nonetheless possible that plasma APOE levels are still relevant to AD pathogenesis. Various studies have demonstrated that *APOE* genotype can influence brain Aβ levels (with *APOE*-ε4 carriers having greater Aβ deposition than non-carriers) [54]–[58] but few have investigated how this relates to plasma APOE levels. Consistent with the various studies just mentioned [54]–[58], one paper on the relationship between *APOE* genotype, brain Aβ deposition and plasma APOE concentration in non-demented individuals [59] reported higher Aβ burden in the medial temporal cortex of *APOE*-ε4 carriers than non-carriers, as assessed by Pittsburgh Compound B retention [59]. However it also reported a positive correlation between plasma APOE concentration and brain Aβ burden, with higher plasma APOE levels in *APOE*-ε4 carriers (*n* = 10) relative to non-carriers (*n* = 29) as measured by ELISA [59]. This is not consistent with several other studies of the relationship between plasma APOE levels and *APOE* genotype [40]–[43] or with our findings in the ADNI cohort. This indicates the need for further research into the relationship between plasma APOE and events in the brain.

Plasma levels of APOE and other proteins may also provide insights into vasculopathy in particular individuals. This may be informative as co-existing vasculopathy can affect AD onset and progression. In this context it is interesting to note that serum glutamic oxaloacetic transaminase, which we identified in signatures discriminating controls from both MCI progressors and AD patients, has been proposed as a predictive biomarker for functional outcome following ischemic stroke [60].

In addition, we cannot exclude the possibility that common polymorphisms in genes other than *APOE* may also affect plasma levels of their corresponding protein or other proteins – this will require further investigation by an integrated analysis of genomic and proteomic data. There could also be effects on levels of plasma proteins due to diet or factors, for example systemic inflammation, which may be affected in various conditions common in older people (e.g. diabetes, heart disease, cancer and arthritis). Such effects may partly account for the large number of inflammatory markers (e.g. interleukins) that were identified in the 18-protein signature of Ray and colleagues but were either not altered or not detectable in the ADNI study.

Another factor that may affect interpretation of the ADNI proteomics data is that, as noted in the ADNI Data Primer, the control samples chosen for proteomic studies were subject to selection bias. Samples selected for inclusion had baseline CSF Aβ_42_ levels above the median baseline CSF Aβ_42_ levels of the control cohort. This led to an abnormally low frequency of the *APOE*-ε4 allele, presumably due to an association between *APOE* genotype and CSF Aβ_42_ levels. While this is an appropriate strategy for improving detectability of differences between the controls and the disease groups, it may have other unanticipated effects, such as those involving plasma APOE levels described above. In addition, the disparity in *APOE*-ε4 frequency is likely to have led to an overestimation of the ability of *APOE* genotype, alone or in combination with demographic variables such as age and gender, to distinguish the clinical groups.

Differences in the size of the participant groups had a profound influence on the values for sensitivity and specificity determined by the classification algorithms. This probably occurs because most classifiers use a training protocol that involves optimizing classification strategies to achieve maximal values for total prediction accuracy. This leads to a bias towards strategies that correctly classify the group with the larger sample size, as this group will constitute a higher proportion of the total sample and will therefore return higher values for total prediction accuracy when called correctly. This highlights one limitation of the plasma analyte dataset currently available for ADNI, which contains considerably fewer control samples than MCI or AD.

Classification strategies that favor sensitivity over specificity are unlikely to be desirable in a clinical diagnostic setting, where it is important to avoid giving healthy people the false impression that they have a terminal disease with no effective treatment. We anticipate that the clinical applicability of the signatures will improve as data become available for larger numbers of cognitively normal controls that are more representative of the general population, allowing more appropriate classification strategies to be selected. Until further control data become available, an alternative approach might be to manually tune classification strategies, based on approaches derived from receiver operating characteristic (ROC) curve analysis, to make specificity a high priority in addition to sensitivity.

The consideration of meta-features representing pair-wise differences generally led to biomarker signatures with improved prediction accuracy relative to signatures comprising single analytes. This may be due to the identification of two analytes that are mathematically or biologically synergistic with regard to disease prediction capacity, the mitigation of confounding effects that arise from inter-sample biological variability or technical variability, or a combination of these factors. The lower accuracy of signatures comprising pair-wise sums probably arises because calculating the sum of Z-score values of *log_10_*-transformed data (comparable to calculating the product of the relative abundance of two analytes) will compound any effects of variability rather than mitigate them.

The meta-feature signatures (Table 5) might also help identify protein interactions of possible biological relevance, as some of the meta-features selected by our method comprise analytes with related molecular functions. Examples of pairs which are potentially related include the chemokine meta-feature pair macrophage inflammatory protein-1α (CCL3) and pulmonary and activation-regulated chemokine (CCL18) and the matrix metalloproteinase-9 and -10 pair. In any event, the improvement in classification accuracy using meta-features demonstrates that the consideration of meta-features represents a useful tool in the search for biomarkers.

While the various signatures proposed here provided accurate classification when considered in the context of the baseline dataset from which they were identified, some performed poorly when tested on data collected at 12 month follow-up. This was particularly true of signatures designed to discriminate controls from individuals with pre-clinical AD (here used to mean individuals with MCI who later progress to AD), in contrast to the signatures designed to discriminate controls from individuals with existing AD, which performed well at both time points. The reasons for this are uncertain. One possible explanation is that if plasma protein profiles reflect the extent of disease progression, it would be expected that protein profiles of controls and AD patients differ more than those of controls and individuals with MCI. As a result, the AD group, being further separated from controls, might be expected to be relatively more robust than the MCI group against fluctuations in plasma analyte levels for any reason.

The ADNI proteomics data that are currently available only cover two time points 12 months apart, a period that may be of insufficient duration to detect substantial clinical or proteomic differences. It would be informative to be able to test our proposed signatures across a wider range of time points if data become available.

Nonetheless, the most accurate classification results we obtained came from signatures that considered the longitudinal change in analyte levels or meta-feature values over the 12 month period. While assessing changes within individuals over time is less convenient than a single test, the stronger performance of a signature based on longitudinal changes suggests that this is an avenue that should be explored in order to improve predictive accuracy.

It was interesting to note the lack of biomarkers that could reliably distinguish individuals with MCI who have progressed to AD from those who have not yet progressed. This is likely to be at least partly due to a high degree of heterogeneity among the non-progressor group. Some might progress to AD in subsequent years, while others may progress to different neurodegenerative conditions. It is also likely that a number of these individuals will remain with only MCI or even revert to control status. It will be important to determine whether the signatures proposed here have the ability to predict which of these individuals will later convert to AD, and we look forward to the outcomes of follow up clinical evaluations.

In addition to unknown endpoints within the MCI group, accurate biomarker identification might also be affected by incorrect ascertainment of AD or co-existing pathologies. As a definitive diagnosis of AD cannot be made until brain pathology is assessed post-mortem, a number of study participants with a clinical diagnosis of AD may have dementias of other etiologies. The extent of co-existing pathologies such as cerebrovasculopathy is also best assessed post-mortem. It will only be possible to select signatures with optimal accuracy for identifying pre-clinical and clinical AD through the use of retrospective analyses after post-mortem pathology has been assessed.

In addition, heterogeneity within the participant groups may also stem from other disease co-morbidities. For example, as noted above, the signatures obtained when considering the longitudinal change from baseline to 12 months follow-up (for both single analytes and meta-features) highlighted a number of analytes that have been reported to have associations with renal failure, heart failure or the metabolic syndrome and diabetes, all of which have been associated with cognitive impairment or AD risk. These conditions are common in older people and may lead to altered plasma protein profiles and a high degree of analyte variation within the participant groups. The classification accuracy of signatures might therefore be improved by first taking into account co-morbidities.

The analyses presented here highlight that in order to identify reliable biomarkers, several factors need to be taken into account. Biomarkers should be robust against age, gender and genotype. In addition, a reliable biomarker should demonstrate minimal variation unrelated to the disease of interest and measurements should not fluctuate substantially between different time points in healthy individuals. These factors may be affected by some of the limitations of the current dataset. For example, as mentioned above, the ADNI control sample was subject to selection bias and so may not accurately represent the parent population. In addition, the Luminex analyte panel used was not designed specifically for AD and better plasma biomarker signatures might be achievable with the measurement of additional components, whether other proteins or non-protein factors such as Aβ, lipids and metals. For example, for CSF biomarkers, combining novel analytes identified using a Luminex proteomics assay with established CSF biomarkers such as Aβ and tau has been shown to substantially improve accuracy in discriminating controls from AD patients [4]. Similar approaches should also be effective with plasma biomarkers. More accurate (although more expensive) diagnostic classification might also be achieved through an integrative approach combining blood and CSF proteomics, genomics and brain imaging.

In conclusion, the findings of this study suggest that sets of plasma analytes can act as useful biomarkers for pre-clinical AD but can be influenced by a number of confounding variables, in particular *APOE* genotype. More research is required on larger samples which allow stratification by potential co-variates while retaining sufficient power for analyses of subgroups. It is likely that plasma biomarkers of the future will involve sets of analytes rather than individual analytes and that accurate pre-clinical diagnosis might require panels of multiple biomarkers. With technological advances in multiplexing protein assays, financial considerations relating to measuring large biomarker panels are becoming less of a barrier to implementation and more importance will instead be placed on assembling optimal panels rather than minimizing the number of proteins.

Furthermore, if costs continue to come down, it may become feasible to perform routine measurements of panels of plasma analytes in ‘at risk’ individuals and monitor the change over time, as is currently done in clinical biochemistry for various markers of health and disease. In addition to providing a cost-effective and minimally-invasive test capable of diagnosing AD in its pre-clinical stages, these approaches may allow us to identify molecular signposts of disease progression, improving understanding of the disease course and facilitating the monitoring of changes over time and responses to interventions.

## Supporting Information

Figure S1 **Heat maps based on the 11-analyte signature generated when (A) including APOE and (B) excluding APOE.** For each analyte in the signature, Z-scores were calculated for all control (*n* = 54) and MCI progressor (*n* = 163) participants. A matrix containing the Z-score values was constructed and rows and columns ordered by similarity, based on the correlation distance, using a Memetic Algorithm (Methods). The output is presented here as a heat map, where samples and analytes with similar ‘expression profiles’ are clustered together. Green indicates lower expression, red indicates higher expression. The bar below the heat map indicates sample class (green – Control; blue – MCI Progressor). Analyte abbreviations are given in Table 3.
(TIF)

Figure S2 **Heat map based on the 6-protein signature generated for** ***APOE*** **-ε3 homozygotes.** The bar below the heat map indicates sample class (green – Control, *n* = 34; blue – MCI Progressor, *n* = 50). Analyte abbreviations are given in Table 3 and Table S6.
(TIF)

Figure S3 **Heat maps based on differences of analyte pairs comprising the signatures generated when (A) including APOE and (B) excluding APOE.** The bar below the heat map indicates sample class (green – Control, *n* = 54; blue – MCI Progressor, *n* = 163). Meta-feature abbreviations are given in Table 5.
(TIF)

Figure S4 **Heat maps based on sums of analyte pairs comprising the signatures generated when (A) including APOE and (B) excluding APOE.** The bar below the heat map indicates sample class (green – Control, *n* = 54; blue – MCI Progressor, *n* = 163). Meta-feature abbreviations are given in Table S8.
(TIF)

Figure S5 **Heat maps based on relative levels of analytes comprising the signature discriminating controls from AD, generated when (A) including APOE and (B) excluding APOE.** The bar below the heat map indicates sample class (green – control, *n* = 54; blue – AD, *n* = 112). Analyte abbreviations are given in Table S11.
(TIF)

Table S1 Set of 10 classifiers used in this study. * Not used for assessment of raw data due to poor performance.
(DOC)

Table S2 Definition of protein abbreviations used in Figure 2.
(DOC)

Table S3 Statistical univariate comparison of plasma analyte levels in Control and MCI Progressor samples. Table lists all analytes that differ significantly (*p*<0.01) in *log_10_* concentration between controls and MCI progressors. Control *n* = 54, MCI Progressor *n* = 163.
(DOC)

Table S4 The 11 analytes that pass the entropy filter when considering sized-matched groups of controls and MCI progressors. * Not selected in either of the 11-analyte signatures generated when considering the full set of samples (Table 3). † Analytes that passed the entropy filter but did not show statistically significant (*p*<0.01) differences between controls and MCI progressors (Table S3). Control *n* = 54, MCI Progressor *n* = 54.
(DOC)

Table S5 Accuracy of analyte signatures from size-matched groups in classifying controls and MCI progressors. Size-matched groups contained 54 controls and 54 MCI progressors. *Sensitivity of the signatures was assessed using a ‘test set’ comprising the remaining 109 MCI progressors.
(DOC)

Table S6 Effect of age and gender on classification accuracy. Size-matched groups were stratified by gender or age (*n* = 27 per group). The Control v MCI Progressor signature was assessed using 10-fold cross-validation.
(DOC)

Table S7 6-analyte signature to discriminate Control and MCI Progressor samples when considering on *APOE*-ε3 homozygous genotypes. Signature was generated using baseline data on the 34 controls and 54 MCI progressors that were homozygous for the *APOE*-ε3 genotype. Italicized analytes were selected in both of the 11-analyte signatures generated from unstratified data (Table 3).
(DOC)

Table S8 Comparison of meta-feature signatures with equally-sized signatures comprising single analytes. ^a^ The full set of samples contained data on 54 controls and 163 MCI progressors. ^b^ The size-matched groups contained data on 54 controls and 54 MCI progressors. For each of these datasets, all samples were used for cross-validation, whereas training and test sets were created by dividing datasets into two equal subsets.
(DOC)

Table S9 Minimal meta-feature set selection of sums of analyte abundances to discriminate Control and MCI Progressor samples. Signatures were generated using baseline data on 54 controls and 163 MCI progressors. Italicized metafeatures contain at least one analyte identified in the corresponding signature generated by single analyte analysis (Table 3).
(DOC)

Table S10 Accuracy of meta-feature signatures involving sums of analyte abundances in classifying controls and MCI progressors. ^a^ The full set of samples contained data on 54 controls and 163 MCI progressors. ^b^ The size-matched groups contained data on 54 controls and 54 MCI progressors. For each of these datasets, all samples were used for cross-validation, whereas training and test sets were created by dividing datasets into two equal subsets.
(DOC)

Table S11 Statistical univariate comparison of plasma analyte levels in Control and AD samples. Table lists all analytes that differ significantly (*p*<0.01) in *log_10_* concentration between controls and AD patients. *Raw data for placenta growth factor were normally distributed and therefore did not require *log_10_* transformation – summary statistics of raw data for placenta growth factor are presented in this table. Control *n* = 54, AD *n* = 112.
(DOC)

Table S12 Feature set selection of signatures* to discriminate Control and AD samples. Signatures were generated using baseline data on 54 controls and 112 AD patients. Italicized analytes were identified in the corresponding signature that best discriminated controls from MCI progressors (Table 3). *Following initial entropy filtering there were several control and AD samples that had the same discretization pattern (i.e. the discrete values across the 13 analytes were identical in a control sample and AD sample – this precludes a solution to the *(α,β)-k-Feature Set problem*). To circumvent this problem for the purpose of generating a solution, the dataset was pruned to remove these samples. Following dataset pruning, 17 analytes passed the entropy filter. These 17 analytes were used to generate the signatures in the table. The pruned samples were incorporated back into the dataset for assessment of classification accuracy.
(DOC)

Table S13 Accuracy of analyte signatures in classifying controls and AD patients. ^a^ The full set of samples contained data on 54 controls and 112 AD patients. ^b^ The size-matched groups contained data on 54 controls and 54 AD patients. *Sensitivity of the signatures was assessed using a ‘test set’ comprising the remaining 58 AD patients.
(DOC)

Table S14 Statistical univariate comparison of plasma analyte levels in MCI Progressor and MCI Other samples. Table lists all analytes that differ significantly (*p*<0.05) in *log_10_* concentration between MCI Progressor and MCI Other groups. MCI Progressor *n* = 163, MCI Other *n* = 233.
(DOC)

Table S15 Previous evidence for altered levels of relevant analytes in the context of AD. The table lists the analytes comprising our 11-analyte signatures (Table 3) that have been investigated in the context of MCI or AD. Unless otherwise stated, the referenced studies of plasma/serum or CSF were consistent with the findings in the ADNI plasma samples. Where findings are inconsistent or a literature search returned no information on levels of particular analytes in plasma/serum or CSF, additional indirect evidence has been cited where available from studies of AD brain or other tissues. To our knowledge, the proteins heparin-binding EGF-like growth factor, betacellulin, brain natriuretic peptide and CD5 have not been investigated in the context of AD.
(DOC)
