# Supplementary figures and images for: Multivariate Protein Signatures of Pre-Clinical Alzheimer's Disease in the Alzheimer's Disease Neuroimaging Initiative (ADNI) Plasma Proteome Dataset
Source: PLoS One. 2012 Apr 2;7(4):e34341. doi: 10.1371/journal.pone.0034341 (PMC3317783; doi:10.1371/journal.pone.0034341)

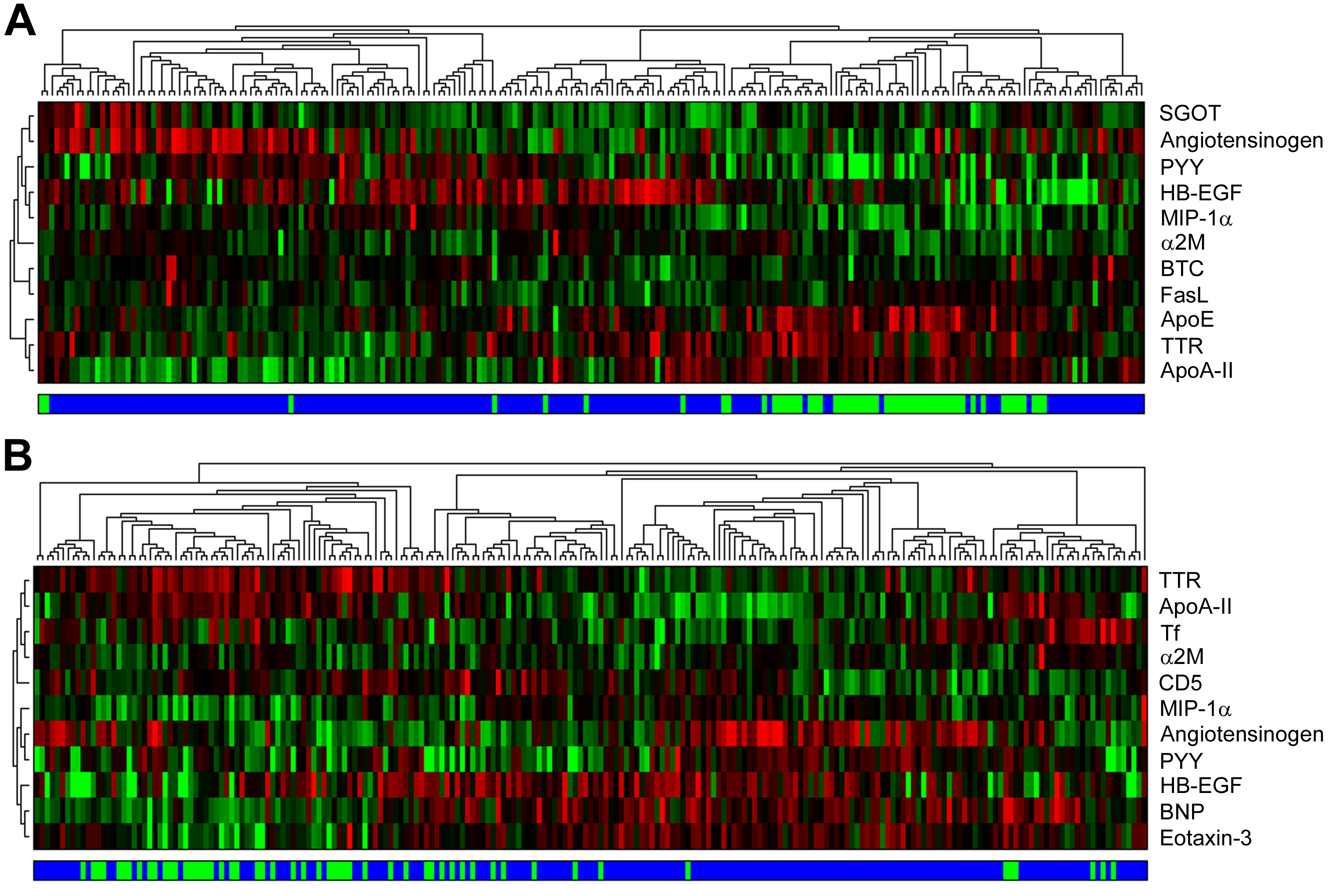

Supplement: Figure S1 — Heat maps based on the 11-analyte signature generated when (A) including APOE and (B) excluding APOE. For each analyte in the signature, Z-scores were calculated for all control (n = 54) and MCI progressor (n = 163) participants. A matrix containing the Z-score values was constructed and rows and columns ordered by similarity, based on the correlation distance, using a Memetic Algorithm (Methods). The output is presented here as a heat map, where samples and analytes with similar ‘expression profiles’ are clustered together. Green indicates lower expression, red indicates higher expression. The bar below the heat map indicates sample class (green – Control; blue – MCI Progressor). Analyte abbreviations are given in Table 3. (TIF) [file pone.0034341.s001.tif]

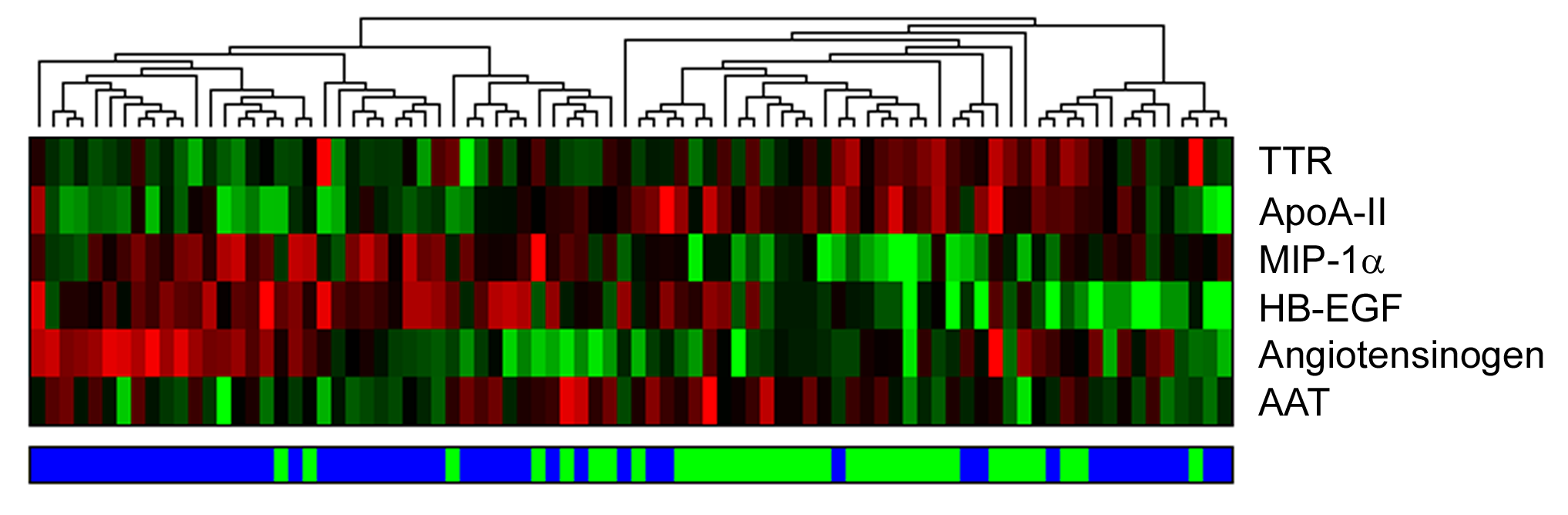

Supplement: Figure S2 — Heat map based on the 6-protein signature generated for APOE -ε3 homozygotes. The bar below the heat map indicates sample class (green – Control, n = 34; blue – MCI Progressor, n = 50). Analyte abbreviations are given in Table 3 and Table S6. (TIF) [file pone.0034341.s002.tif]

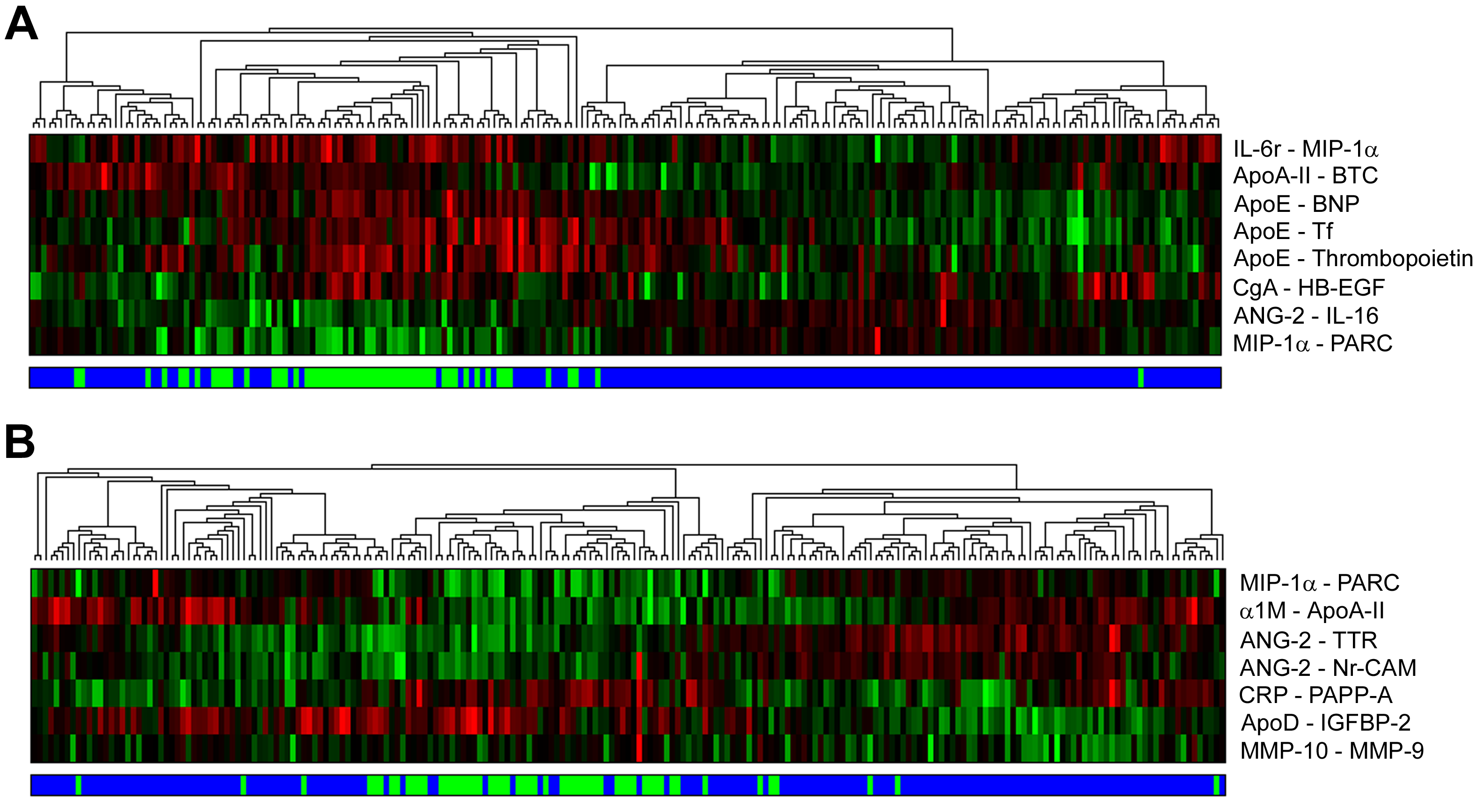

Supplement: Figure S3 — Heat maps based on differences of analyte pairs comprising the signatures generated when (A) including APOE and (B) excluding APOE. The bar below the heat map indicates sample class (green – Control, n = 54; blue – MCI Progressor, n = 163). Meta-feature abbreviations are given in Table 5. (TIF) [file pone.0034341.s003.tif]

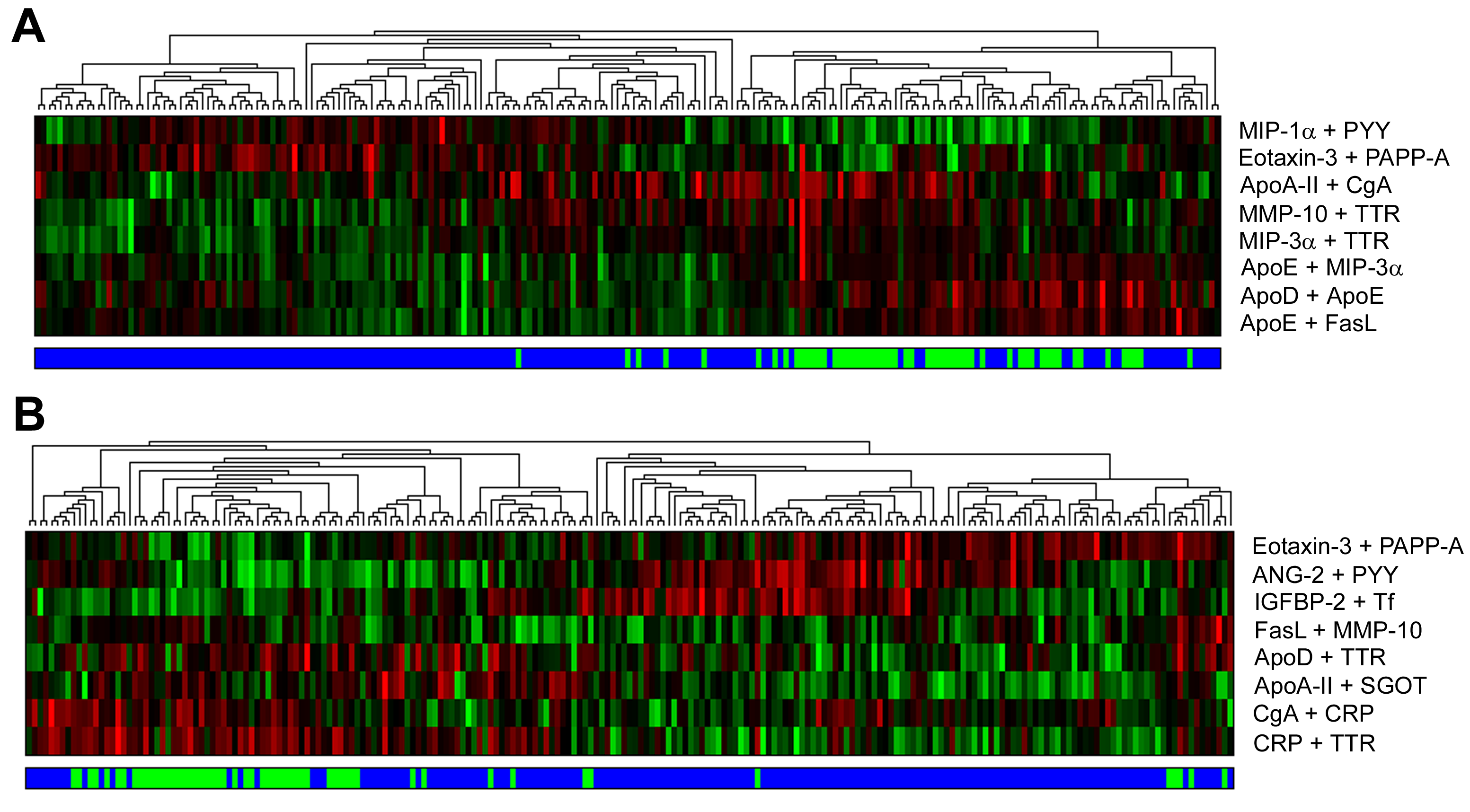

Supplement: Figure S4 — Heat maps based on sums of analyte pairs comprising the signatures generated when (A) including APOE and (B) excluding APOE. The bar below the heat map indicates sample class (green – Control, n = 54; blue – MCI Progressor, n = 163). Meta-feature abbreviations are given in Table S8. (TIF) [file pone.0034341.s004.tif]

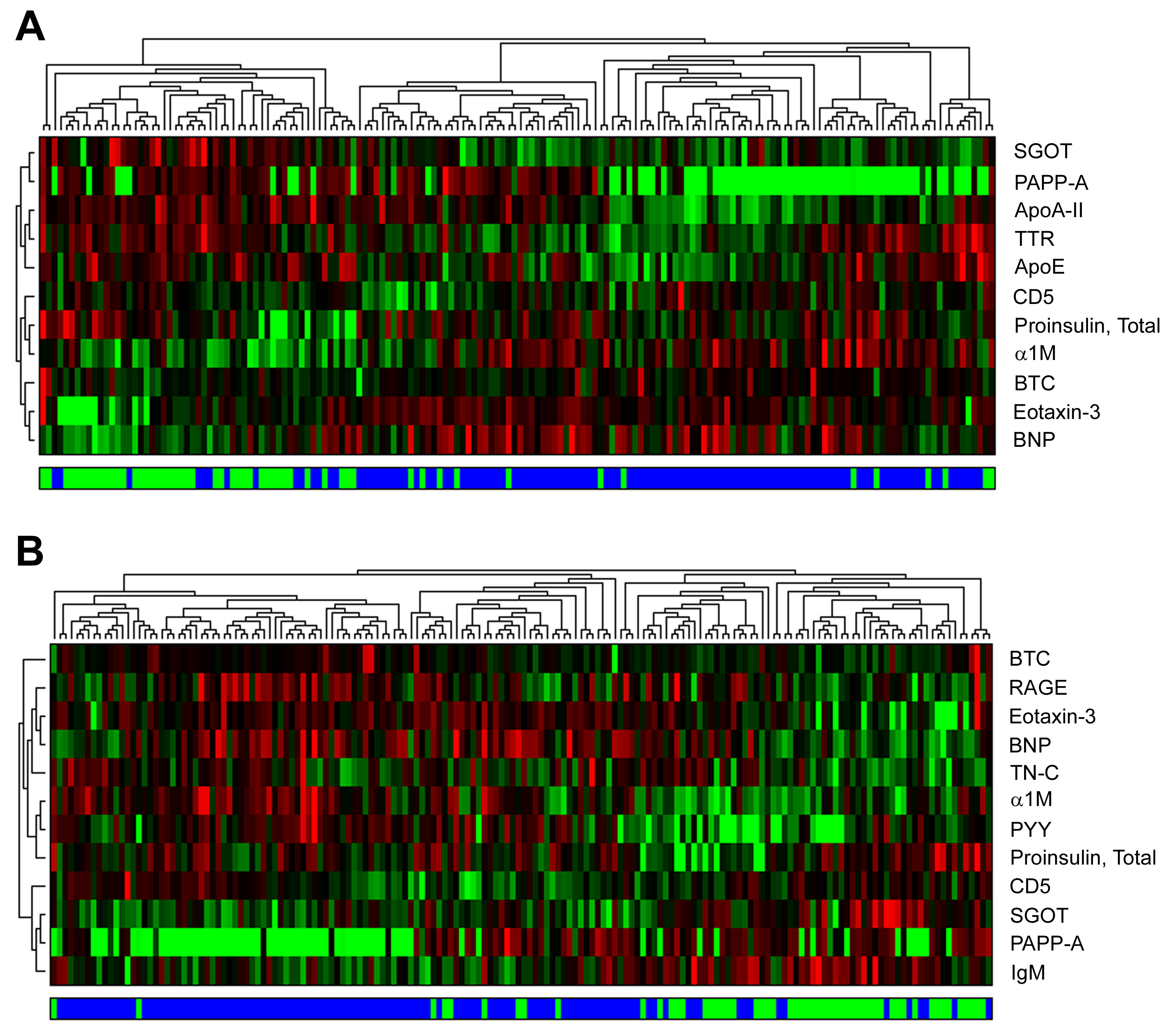

Supplement: Figure S5 — Heat maps based on relative levels of analytes comprising the signature discriminating controls from AD, generated when (A) including APOE and (B) excluding APOE. The bar below the heat map indicates sample class (green – control, n = 54; blue – AD, n = 112). Analyte abbreviations are given in Table S11. (TIF) [file pone.0034341.s005.tif]
