# Supplementary material for: Multivariate Protein Signatures of Pre-Clinical Alzheimer's Disease in the Alzheimer's Disease Neuroimaging Initiative (ADNI) Plasma Proteome Dataset
Source: PLoS One. 2012 Apr 2;7(4):e34341. doi: 10.1371/journal.pone.0034341 (PMC3317783; doi:10.1371/journal.pone.0034341)
Supplement: Table S1 — Set of 10 classifiers used in this study. * Not used for assessment of raw data due to poor performance. (DOC) [file pone.0034341.s006.doc]

Table S1. Set of 10 classifiers used in this study.

| **Classifier** | **Learning Approach** |
| --- | --- |
| BayesNet | Bayes |
| *NaiveBayes | Bayes |
| *MultilayerPerceptron | Functions |
| RBFNetwork | Functions |
| AdaBoostM1 | Meta |
| LogitBoost | Meta |
| RandomCommittee | Meta |
| RotationForest | Meta |
| ADTree | Trees |
| RandomForest | Trees |

* Not used for assessment of raw data due to poor performance.
