# Supplementary material for: Multivariate Protein Signatures of Pre-Clinical Alzheimer's Disease in the Alzheimer's Disease Neuroimaging Initiative (ADNI) Plasma Proteome Dataset
Source: PLoS One. 2012 Apr 2;7(4):e34341. doi: 10.1371/journal.pone.0034341 (PMC3317783; doi:10.1371/journal.pone.0034341)
Supplement: Table S2 — Definition of protein abbreviations used in Figure 2. (DOC) [file pone.0034341.s007.doc]

Table S2. Definition of protein abbreviations used in Figure 2.

| ANG-2 | Angiopoietin 2 |
| --- | --- |
| EGF | Epidermal growth factor |
| G-CSF | Granulocyte colony stimulating factor |
| GDNF | Glial cell-derived neurotrophic factor |
| ICAM-1 | Intercellular adhesion molecule 1 |
| IGFBP-6 | Insulin-like growth factor-binding protein 6 |
| IL-1α | Interleukin 1α |
| IL-3 | Interleukin 3 |
| IL-8 | Interleukin 8 |
| IL-11 | Interleukin 11 |
| M-CSF | Macrophage colony stimulating factor |
| MCP-3 | Monocyte chemotactic protein 3 |
| MIP-1δ | Macrophage inflammatory protein 1δ |
| PDGF-BB | Platelet-derived growth factor BB |
| PARC | Pulmonary and activation-related chemokine |
| RANTES | T-cell-specific protein RANTES |
| TNF-α | Tumor necrosis factor α |
| TRAIL-R4 | Tumor necrosis factor receptor superfamily, member 10d, decoy with truncated death domain |
