# Supplementary material for: Multivariate Protein Signatures of Pre-Clinical Alzheimer's Disease in the Alzheimer's Disease Neuroimaging Initiative (ADNI) Plasma Proteome Dataset
Source: PLoS One. 2012 Apr 2;7(4):e34341. doi: 10.1371/journal.pone.0034341 (PMC3317783; doi:10.1371/journal.pone.0034341)
Supplement: Table S3 — Statistical univariate comparison of plasma analyte levels in Control and MCI Progressor samples. Table lists all analytes that differ significantly (p<0.01) in log10 concentration between controls and MCI progressors. Control n = 54, MCI Progressor n = 163. (DOC) [file pone.0034341.s008.doc]

Table S3. Statistical univariate comparison of plasma analyte levels in Control and MCI Progressor samples.

| **Analyte (units measured)** | **Mean log_10_ concentration (SEM)** | | ***p* value** |
| --- | --- | --- | --- |
|  | **Control** | **MCI Progressor** |  |
| Apolipoprotein E (µg/ml) | 1.853 (0.020) | 1.652 (0.014) | 2.7x10^-13^ |
| Apolipoprotein A-II (ng/ml) | 2.762 (0.011) | 2.683 (0.009) | 4.5x10^-8^ |
| Transthyretin (mg/dl) | 2.617 (0.012) | 2.547 (0.008) | 2.6x10^-6^ |
| Macrophage Inflammatory Protein-1α (pg/ml) | 2.167 (0.015) | 2.240 (0.009) | 4.0x10^-5^ |
| Brain Natriuretic Peptide (pg/ml) | 2.779 (0.054) | 3.016 (0.024) | 1.3x10^-4^ |
| Peptide YY (pg/ml) | 1.840 (0.042) | 2.027 (0.022) | 1.6x10^-4^ |
| C-Reactive Protein (µg/ml) | 0.305 (0.065) | 0.010 (0.037) | 1.6x10^-4^ |
| Angiotensinogen (ng/ml) | 1.016 (0.102) | 1.487 (0.064) | 1.7x10^-4^ |
| Insulin-like Growth Factor-Binding Protein 2 (ng/ml) | 1.894 (0.026) | 2.019 (0.019) | 2.1x10^-4^ |
| Eotaxin-3 (pg/ml) | 2.376 (0.059) | 2.613 (0.018) | 2.6x10^-4^ |
| Immunoglobulin M (mg/ml) | 0.376 (0.032) | 0.239 (0.021) | 5.2x10^-4^ |
| Interleukin-16 (pg/ml) | 2.603 (0.017) | 2.525 (0.014) | 5.7x10^-4^ |
| Pregnancy-Associated Plasma Protein A (mIU/ml) | -1.796 (0.039) | -1.648 (0.020) | 0.0010 |
| Heparin-Binding EGF-Like Growth Factor (pg/ml) | 1.668 (0.048) | 1.861 (0.031) | 0.0011 |
| Pulmonary and Activation-Regulated Chemokine (ng/ml) | 2.104 (0.023) | 2.016 (0.012) | 0.0011 |
| Angiopoietin-2 (ng/ml) | 0.596 (0.021) | 0.677 (0.012) | 0.0011 |
| Fas Ligand (pg/ml) | 1.849 (0.028) | 1.738 (0.021) | 0.0018 |
| Neuronal Cell Adhesion Molecule (ng/ml) | 0.402 (0.016) | 0.331 (0.016) | 0.0020 |
| Interleukin-6 receptor (ng/ml) | 1.508 (0.016) | 1.446 (0.011) | 0.0022 |
| Apolipoprotein D (µg/ml) | 2.237 (0.016) | 2.180 (0.010) | 0.0033 |
| Monokine Induced by Gamma Interferon (pg/ml) | 3.612 (0.033) | 3.503 (0.017) | 0.0042 |
| CD5 (ng/ml) | 3.578 (0.017) | 3.521 (0.012) | 0.0071 |
| Pancreatic Polypeptide (pg/ml) | 1.992 (0.043) | 2.133 (0.028) | 0.0071 |
| Alpha-1-Microglobulin (µg/ml) | 1.034 (0.015) | 1.081 (0.009) | 0.0080 |
| Leptin (ng/ml) | 1.099 (0.058) | 0.926 (0.030) | 0.0095 |

Table lists all analytes that differ significantly (*p*<0.01) in *log_10_* concentration between controls and MCI progressors. Control *n*=54, MCI Progressor *n*=163.
