# Supplementary material for: Multivariate Protein Signatures of Pre-Clinical Alzheimer's Disease in the Alzheimer's Disease Neuroimaging Initiative (ADNI) Plasma Proteome Dataset
Source: PLoS One. 2012 Apr 2;7(4):e34341. doi: 10.1371/journal.pone.0034341 (PMC3317783; doi:10.1371/journal.pone.0034341)
Supplement: Table S4 — The 11 analytes that pass the entropy filter when considering sized-matched groups of controls and MCI progressors. * Not selected in either of the 11-analyte signatures generated when considering the full set of samples (Table 3). † Analytes that passed the entropy filter but did not show statistically significant (p<0.01) differences between controls and MCI progressors (Table S3). Control n = 54, MCI Progressor n = 54. (DOC) [file pone.0034341.s009.doc]

Table S4. The 11 analytes that pass the entropy filter when considering sized-matched groups of controls and MCI progressors.

| **Protein Name** |
| --- |
| Apolipoprotein A-II |
| Apolipoprotein E |
| † Betacellulin |
| Brain Natriuretic Peptide |
| CD5 |
| Eotaxin-3 |
| Heparin-Binding EGF-Like Growth Factor |
| Macrophage Inflammatory Protein-1α |
| † Serum Glutamic Oxaloacetic Transaminase |
| *† Tamm-Horsfall Urinary Glycoprotein |
| Transthyretin |

* Not selected in either of the 11-analyte signatures generated when considering the full set of samples (Table 3). † Analytes that passed the entropy filter but did not show statistically significant (*p*<0.01) differences between controls and MCI progressors (Table S3). Control *n*=54, MCI Progressor *n*=54.
