# Supplementary material for: Multivariate Protein Signatures of Pre-Clinical Alzheimer's Disease in the Alzheimer's Disease Neuroimaging Initiative (ADNI) Plasma Proteome Dataset
Source: PLoS One. 2012 Apr 2;7(4):e34341. doi: 10.1371/journal.pone.0034341 (PMC3317783; doi:10.1371/journal.pone.0034341)
Supplement: Table S5 — Accuracy of analyte signatures from size-matched groups in classifying controls and MCI progressors. Size-matched groups contained 54 controls and 54 MCI progressors. *Sensitivity of the signatures was assessed using a ‘test set’ comprising the remaining 109 MCI progressors. (DOC) [file pone.0034341.s010.doc]

Table S5. Accuracy of analyte signatures from size-matched groups in classifying controls and MCI progressors.

| **Signature** | **Cross-Validation** | | ***Test Set** | |
| --- | --- | --- | --- | --- |
|  | Sens | Spec | Sens | Spec |
| 11-analyte signature on size-matched groups with APOE | 71.7 | 76.7 | 85.7 | N/A |
| 10-analyte signature on size-matched groups without APOE | 66.9 | 75.4 | 76.6 | N/A |

Size-matched groups contained 54 controls and 54 MCI progressors.
*Sensitivity of the signatures was assessed using a ‘test set’ comprising the remaining 109 MCI progressors.
