# Supplementary material for: Multivariate Protein Signatures of Pre-Clinical Alzheimer's Disease in the Alzheimer's Disease Neuroimaging Initiative (ADNI) Plasma Proteome Dataset
Source: PLoS One. 2012 Apr 2;7(4):e34341. doi: 10.1371/journal.pone.0034341 (PMC3317783; doi:10.1371/journal.pone.0034341)
Supplement: Table S6 — Effect of age and gender on classification accuracy. Size-matched groups were stratified by gender or age (n = 27 per group). The Control v MCI Progressor signature was assessed using 10-fold cross-validation. (DOC) [file pone.0034341.s011.doc]

Table S6. Effect of age and gender on classification accuracy.

| **Signature** | **Sens** | **Spec** | **MCC** |
| --- | --- | --- | --- |
| Signature with APOE  *Female*  *Male* | 73.7  74.8 | 76.3  76.7 | 0.50  0.52 |
| Signature without APOE  *Female*  *Male* | 72.6  76.7 | 75.2  70.4 | 0.48  0.47 |
| Signature with APOE  *Young*  *Old* | 64.4  74.1 | 72.6  76.7 | 0.37  0.51 |
| Signature without APOE  *Young*  *Old* | 71.9  72.6 | 73.3  76.7 | 0.46  0.49 |

Size-matched groups were stratified by gender or age (*n*=27 per group). The Control v MCI Progressor signature was assessed using 10-fold cross-validation.
