# Supplementary material for: Multivariate Protein Signatures of Pre-Clinical Alzheimer's Disease in the Alzheimer's Disease Neuroimaging Initiative (ADNI) Plasma Proteome Dataset
Source: PLoS One. 2012 Apr 2;7(4):e34341. doi: 10.1371/journal.pone.0034341 (PMC3317783; doi:10.1371/journal.pone.0034341)
Supplement: Table S7 — 6-analyte signature to discriminate Control and MCI Progressor samples when considering on APOE-ε3 homozygous genotypes. Signature was generated using baseline data on the 34 controls and 54 MCI progressors that were homozygous for the APOE-ε3 genotype. Italicized analytes were selected in both of the 11-analyte signatures generated from unstratified data (Table 3). (DOC) [file pone.0034341.s012.doc]

Table S7. 6-analyte signature to discriminate Control and MCI Progressor samples when considering on *APOE*-ε3 homozygous genotypes.

| **Analyte (abbreviation)** |
| --- |
| Alpha-1-Antitrypsin (AAT) |
| *Angiotensinogen* |
| *Apolipoprotein A-II* |
| *Heparin-Binding EGF-Like Growth Factor* |
| *Macrophage Inflammatory Protein-1α* |
| *Transthyretin* |

Signature was generated using baseline data on the 34 controls and 54 MCI progressors that were homozygous for the *APOE*-ε3 genotype. Italicized analytes were selected in both of the 11-analyte signatures generated from unstratified data (Table 3).
