# Supplementary material for: Multivariate Protein Signatures of Pre-Clinical Alzheimer's Disease in the Alzheimer's Disease Neuroimaging Initiative (ADNI) Plasma Proteome Dataset
Source: PLoS One. 2012 Apr 2;7(4):e34341. doi: 10.1371/journal.pone.0034341 (PMC3317783; doi:10.1371/journal.pone.0034341)
Supplement: Table S8 — Comparison of meta-feature signatures with equally-sized signatures comprising single analytes. a The full set of samples contained data on 54 controls and 163 MCI progressors. b The size-matched groups contained data on 54 controls and 54 MCI progressors. For each of these datasets, all samples were used for cross-validation, whereas training and test sets were created by dividing datasets into two equal subsets. (DOC) [file pone.0034341.s013.doc]

Table S8. Comparison of meta-feature signatures with equally-sized signatures comprising single analytes.

| **Signature** | **Cross-Validation** | | | **Training Set** | | | **Test Set** | | |
| --- | --- | --- | --- | --- | --- | --- | --- | --- | --- |
|  | Sens | Spec | MCC | Sens | Spec | MCC | Sens | Spec | MCC |
| 8-metafeature signature with APOE |  |  |  |  |  |  |  |  |  |
| ^a^*Full set of samples* | 94.7 | 78.1 | 0.74 | 99.1 | 96.3 | 0.96 | 93.2 | 64.8 | 0.61 |
| ^b^*Size-matched groups* | 90.2 | 87.2 | 0.77 | 98.9 | 98.1 | 0.97 | 85.6 | 86.7 | 0.73 |
| 7-metafeature signature without APOE |  |  |  |  |  |  |  |  |  |
| *Full set of samples* | 95.6 | 78.1 | 0.76 | 97.9 | 95.9 | 0.93 | 96.0 | 72.6 | 0.73 |
| *Size-matched groups* | 83.3 | 87.6 | 0.71 | 90.7 | 99.2 | 0.91 | 90.7 | 83.0 | 0.74 |
| Top 13 single analytes - Feature Set selection |  |  |  |  |  |  |  |  |  |
| *Full set of samples* | 93.4 | 62.6 | 0.60 | 97.9 | 91.5 | 0.90 | 94.0 | 53.7 | 0.54 |
| *Size-matched groups* | 76.7 | 76.5 | 0.54 | 97.4 | 94.8 | 0.92 | 79.6 | 72.6 | 0.53 |
| Top 13 single analytes - *p* value |  |  |  |  |  |  |  |  |  |
| *Full set of samples* | 91.5 | 59.4 | 0.54 | 97.6 | 93.0 | 0.91 | 92.3 | 58.9 | 0.55 |
| *Size-matched groups* | 69.6 | 68.0 | 0.38 | 91.1 | 95.6 | 0.87 | 83.3 | 64.4 | 0.49 |

^a^ The full set of samples contained data on 54 controls and 163 MCI progressors. ^b^ The size-matched groups contained data on 54 controls and 54 MCI progressors. For each of these datasets, all samples were used for cross-validation, whereas training and test sets were created by dividing datasets into two equal subsets.
