# Supplementary material for: Multivariate Protein Signatures of Pre-Clinical Alzheimer's Disease in the Alzheimer's Disease Neuroimaging Initiative (ADNI) Plasma Proteome Dataset
Source: PLoS One. 2012 Apr 2;7(4):e34341. doi: 10.1371/journal.pone.0034341 (PMC3317783; doi:10.1371/journal.pone.0034341)
Supplement: Table S9 — Minimal meta-feature set selection of sums of analyte abundances to discriminate Control and MCI Progressor samples. Signatures were generated using baseline data on 54 controls and 163 MCI progressors. Italicized metafeatures contain at least one analyte identified in the corresponding signature generated by single analyte analysis (Table 3). (DOC) [file pone.0034341.s014.doc]

Table S9. Minimal meta-feature set selection of sums of analyte abundances to discriminate Control and MCI Progressor samples.

| **Analyte pairs (abbreviation)** |  |
| --- | --- |
| **Including APOE** | **Excluding APOE** |
| *Apolipoprotein A-II & Chromogranin-A (ApoA-II + CgA)* | *Angiopoietin-2 & Peptide YY  (ANG-2 + PPY)* |
| *Apolipoprotein D & Apolipoprotein E (ApoD + ApoE)* | *Apolipoprotein A-II & Serum Glutamic Oxaloacetic Transaminase  (ApoA-II + SGOT)* |
| *Apolipoprotein E & Fas Ligand  (ApoE + FasL)* | *Apolipoprotein D & Transthyretin  (ApoD + TTR)* |
| *Apolipoprotein E & Macrophage Inflammatory Protein-3α (ApoE + MIP-3α)* | *C-Reactive Protein & Transthyretin  (CRP + TTR)* |
| Eotaxin-3 & Pregnancy-Associated Plasma Protein A (Eotaxin-3 + PAPP-A) | Chromogranin-A & C-Reactive Protein  (CgA + CRP) |
| *Macrophage Inflammatory Protein-1α & Peptide YY (MIP-1α + PYY)* | *Eotaxin-3 & Pregnancy-Associated Plasma Protein A (Eotaxin-3 + PAPP-A)* |
| *Macrophage Inflammatory Protein-3α & Transthyretin (MIP-3α + TTR)* | Fas Ligand & Matrix Metalloproteinase-10 (FasL + MMP-10) |
| *Matrix Metalloproteinase-10 & Transthyretin (MMP-10 + TTR)* | *Insulin-like Growth Factor-Binding Protein 2 & Serotransferrin (IGFBP-2 + Tf)* |

Signatures were generated using baseline data on 54 controls and 163 MCI progressors. Italicized metafeatures contain at least one analyte identified in the corresponding signature generated by single analyte analysis (Table 3).
