# Supplementary material for: Multivariate Protein Signatures of Pre-Clinical Alzheimer's Disease in the Alzheimer's Disease Neuroimaging Initiative (ADNI) Plasma Proteome Dataset
Source: PLoS One. 2012 Apr 2;7(4):e34341. doi: 10.1371/journal.pone.0034341 (PMC3317783; doi:10.1371/journal.pone.0034341)
Supplement: Table S10 — Accuracy of meta-feature signatures involving sums of analyte abundances in classifying controls and MCI progressors. a The full set of samples contained data on 54 controls and 163 MCI progressors. b The size-matched groups contained data on 54 controls and 54 MCI progressors. For each of these datasets, all samples were used for cross-validation, whereas training and test sets were created by dividing datasets into two equal subsets. (DOC) [file pone.0034341.s015.doc]

Table S10. Accuracy of meta-feature signatures involving sums of analyte abundances in classifying controls and MCI progressors.

| **Signature** | **Cross-Validation** | | **Training Set** | | **Test Set** | |
| --- | --- | --- | --- | --- | --- | --- |
|  | Sens | Spec | Sens | Spec | Sens | Spec |
| 8-metafeature signature with APOE |  |  |  |  |  |  |
| ^a^*Full set of samples* | 93.4 | 69.1 | 97.1 | 92.2 | 91.2 | 65.2 |
| ^b^*Size-matched groups* | 76.7 | 77.2 | 97.0 | 94.1 | 79.6 | 67.4 |
| 8-metafeature signature without APOE |  |  |  |  |  |  |
| *Full set of samples* | 93.8 | 71.1 | 99.1 | 95.9 | 93.2 | 58.1 |
| *Size-matched groups* | 73.9 | 80.4 | 94.8 | 94.8 | 74.8 | 69.6 |

^a^ The full set of samples contained data on 54 controls and 163 MCI progressors. ^b^ The size-matched groups contained data on 54 controls and 54 MCI progressors. For each of these datasets, all samples were used for cross-validation, whereas training and test sets were created by dividing datasets into two equal subsets.
