# Supplementary material for: Multivariate Protein Signatures of Pre-Clinical Alzheimer's Disease in the Alzheimer's Disease Neuroimaging Initiative (ADNI) Plasma Proteome Dataset
Source: PLoS One. 2012 Apr 2;7(4):e34341. doi: 10.1371/journal.pone.0034341 (PMC3317783; doi:10.1371/journal.pone.0034341)
Supplement: Table S11 — Statistical univariate comparison of plasma analyte levels in Control and AD samples. Table lists all analytes that differ significantly (p<0.01) in log10 concentration between controls and AD patients. *Raw data for placenta growth factor were normally distributed and therefore did not require log10 transformation – summary statistics of raw data for placenta growth factor are presented in this table. Control n = 54, AD n = 112. (DOC) [file pone.0034341.s016.doc]

Table S11. Statistical univariate comparison of plasma analyte levels in Control and AD samples.

| **Analyte (units measured)** | **Mean log_10_ concentration (SEM)** | | ***p* value** |
| --- | --- | --- | --- |
|  | **Control** | **AD** |  |
| Apolipoprotein E (µg/ml) | 1.853 (0.020) | 1.712 (0.018) | 5.2x10^-7^ |
| Apolipoprotein A-II (ng/ml) | 2.762 (0.011) | 2.689 (0.009) | 8.7x10^-7^ |
| Immunoglobulin M (mg/ml) | 0.376 (0.032) | 0.187 (0.023) | 6.3x10^-6^ |
| Serum Glutamic Oxaloacetic Transaminase (µg/ml) | 3.701 (0.137) | 2.950 (0.076) | 6.4x10^-6^ |
| Peptide YY (pg/ml) | 1.840 (0.042) | 2.075 (0.030) | 1.3x10^-5^ |
| Alpha-1-Microglobulin (µg/ml) | 1.034 (0.015) | 1.115 (0.010) | 1.4x10^-5^ |
| Brain Natriuretic Peptide (pg/ml) | 2.779 (0.054) | 3.054 (0.031) | 2.6x10^-5^ |
| *Placenta Growth Factor (pg/ml) | 36.54 (1.436) | 27.99 (1.418) | 4.1x10^-5^ |
| Eotaxin-3 (pg/ml) | 2.376 (0.059) | 2.618 (0.020) | 0.0002 |
| Tissue Inhibitor of Metalloproteinases 1 (ng/ml) | 2.004 (0.013) | 2.066 (0.011) | 0.0004 |
| Pregnancy-Associated Plasma Protein A (mIU/ml) | -1.796 (0.039) | -1.971 (0.032) | 0.0006 |
| Transthyretin (mg/dl) | 2.617 (0.012) | 2.560 (0.012) | 0.0009 |
| Interleukin-16 (pg/ml) | 2.603 (0.017) | 2.537 (0.012) | 0.0023 |
| Vitronectin (µg/ml) | 2.976 (0.008) | 2.943 (0.008) | 0.0034 |
| Tenascin-C (ng/ml) | 2.688 (0.027) | 2.783 (0.017) | 0.0040 |
| Neutrophil Gelatinase-Associated Lipocalin (ng/ml) | 2.403 (0.015) | 2.462 (0.009) | 0.0040 |
| Vascular Cell Adhesion Molecule-1 (ng/ml) | 2.843 (0.014) | 2.895 (0.011) | 0.0044 |
| Pancreatic Polypeptide (pg/ml) | 1.992 (0.043) | 2.152 (0.038) | 0.0061 |
| Alpha-2-Macroglobulin (mg/ml) | 0.028 (0.015) | 0.075 (0.009) | 0.0068 |
| Tamm-Horsfall Urinary Glycoprotein (µg/ml) | -1.329 (0.024) | -1.410 (0.017) | 0.0069 |
| Heparin-Binding EGF-Like Growth Factor (pg/ml) | 1.668 (0.048) | 1.819 (0.027) | 0.0071 |

Table lists all analytes that differ significantly (*p*<0.01) in *log_10_* concentration between controls and AD patients. *Raw data for placenta growth factor were normally distributed and therefore did not require *log_10_* transformation – summary statistics of raw data for placenta growth factor are presented in this table. Control *n*=54, AD *n*=112.
