# Supplementary material for: Multivariate Protein Signatures of Pre-Clinical Alzheimer's Disease in the Alzheimer's Disease Neuroimaging Initiative (ADNI) Plasma Proteome Dataset
Source: PLoS One. 2012 Apr 2;7(4):e34341. doi: 10.1371/journal.pone.0034341 (PMC3317783; doi:10.1371/journal.pone.0034341)
Supplement: Table S12 — Feature set selection of signatures* to discriminate Control and AD samples. Signatures were generated using baseline data on 54 controls and 112 AD patients. Italicized analytes were identified in the corresponding signature that best discriminated controls from MCI progressors (Table 3). *Following initial entropy filtering there were several control and AD samples that had the same discretization pattern (i.e. the discrete values across the 13 analytes were identical in a control sample and AD sample – this precludes a solution to the (α,β)-k-Feature Set problem). To circumvent this problem for the purpose of generating a solution, the dataset was pruned to remove these samples. Following dataset pruning, 17 analytes passed the entropy filter. These 17 analytes were used to generate the signatures in the table. The pruned samples were incorporated back into the dataset for assessment of classification accuracy. (DOC) [file pone.0034341.s017.doc]

Table S12. Feature set selection of signatures* to discriminate Control and AD samples

| **Analyte (abbreviation)** |  |
| --- | --- |
| **a) Including APOE** | **b) Excluding APOE** |
| α1-Microglobulin (α1M) | α1-Microglobulin |
| *Apolipoprotein A-II (ApoA-II)* | Betacellulin |
| *Apolipoprotein E (ApoE)* | *Brain Natriuretic Peptide* |
| *Betacellulin (BTC)* | *CD5* |
| Brain Natriuretic Peptide (BNP) | *Eotaxin-3* |
| CD5 | Immunoglobulin M (IgM) |
| Eotaxin-3 | *Peptide YY (PYY)* |
| Pregnancy-Associated Plasma Protein A (PAPP-A) | Pregnancy-Associated Plasma Protein A |
| Proinsulin-Total | Proinsulin-Total |
| *Serum Glutamic Oxaloacetic Transaminase (SGOT)* | Receptor for advanced glycosylation end product (RAGE) |
| *Transthyretin (TTR)* | Serum Glutamic Oxaloacetic Transaminase |
|  | Tenascin-C (TN-C) |

Signatures were generated using baseline data on 54 controls and 112 AD patients. Italicized analytes were identified in the corresponding signature that best discriminated controls from MCI progressors (Table 3).

*Following initial entropy filtering there were several control and AD samples that had the same discretization pattern (i.e. the discrete values across the 13 analytes were identical in a control sample and AD sample – this precludes a solution to the *(α,β)-k-Feature Set problem*). To circumvent this problem for the purpose of generating a solution, the dataset was pruned to remove these samples. Following dataset pruning, 17 analytes passed the entropy filter. These 17 analytes were used to generate the signatures in the table. The pruned samples were incorporated back into the dataset for assessment of classification accuracy.
