# Supplementary material for: Multivariate Protein Signatures of Pre-Clinical Alzheimer's Disease in the Alzheimer's Disease Neuroimaging Initiative (ADNI) Plasma Proteome Dataset
Source: PLoS One. 2012 Apr 2;7(4):e34341. doi: 10.1371/journal.pone.0034341 (PMC3317783; doi:10.1371/journal.pone.0034341)
Supplement: Table S13 — Accuracy of analyte signatures in classifying controls and AD patients. a The full set of samples contained data on 54 controls and 112 AD patients. b The size-matched groups contained data on 54 controls and 54 AD patients. *Sensitivity of the signatures was assessed using a ‘test set’ comprising the remaining 58 AD patients. (DOC) [file pone.0034341.s018.doc]

Table S13. Accuracy of analyte signatures in classifying controls and AD patients.

| **Signature** | **Cross-Validation** | | ***Test Set** | |
| --- | --- | --- | --- | --- |
|  | Sens | Spec | Sens | Spec |
| 11-analyte signature with APOE |  |  |  |  |
| ^a^*Full set of samples* | 86.1 | 64.6 | N/A | N/A |
| ^b^*Size-matched groups* | 73.7 | 73.1 | 84.3 | N/A |
| 12-analyte signature without APOE |  |  |  |  |
| *Full set of samples* | 85.8 | 60.2 | N/A | N/A |
| *Size-matched groups* | 67.4 | 67.8 | 79.5 | N/A |

^a^ The full set of samples contained data on 54 controls and 112 AD patients. ^b^ The size-matched groups contained data on 54 controls and 54 AD patients.

*Sensitivity of the signatures was assessed using a ‘test set’ comprising the remaining 58 AD patients.
