# Supplementary material for: Multivariate Protein Signatures of Pre-Clinical Alzheimer's Disease in the Alzheimer's Disease Neuroimaging Initiative (ADNI) Plasma Proteome Dataset
Source: PLoS One. 2012 Apr 2;7(4):e34341. doi: 10.1371/journal.pone.0034341 (PMC3317783; doi:10.1371/journal.pone.0034341)
Supplement: Table S14 — Statistical univariate comparison of plasma analyte levels in MCI Progressor and MCI Other samples. Table lists all analytes that differ significantly (p<0.05) in log10 concentration between MCI Progressor and MCI Other groups. MCI Progressor n = 163, MCI Other n = 233. (DOC) [file pone.0034341.s019.doc]

Table S14. Statistical univariate comparison of plasma analyte levels in MCI Progressor and MCI Other samples.

| **Analyte (units measured)** | **Mean log_10_ concentration (SEM)** | | ***p* value** |
| --- | --- | --- | --- |
|  | **MCI Other** | **MCI Progressor** |  |
| Macrophage Inflammatory Protein-3α | 1.821 (0.016) | 1.753 (0.014) | 0.0015 |
| Cortisol | 2.138 (0.008) | 2.181 (0.011) | 0.0018 |
| Apolipoprotein E | 1.704 (0.013) | 1.652 (0.014) | 0.0054 |
| C-Reactive Protein | 0.148 (0.034) | 0.011 (0.037) | 0.0065 |
| Serum Amyloid P-Component | 1.335 (0.008) | 1.301 (0.010) | 0.0074 |
| Proinsulin (Total) | 1.202 (0.022) | 1.121 (0.024) | 0.0138 |
| Pulmonary and Activation-Regulated Chemokine | 2.052 (0.011) | 2.015 (0.012) | 0.0244 |
| Prostatic Acid Phosphatase | -0.701 (0.016) | -0.750 (0.016) | 0.0263 |
| Proinsulin (Intact) | 0.605 (0.022) | 0.540 (0.023) | 0.0418 |

Table lists all analytes that differ significantly (*p*<0.05) in *log_10_* concentration between MCI Progressor and MCI Other groups. MCI Progressor *n*=163, MCI Other *n*=233.
