# Supplementary material for: Multivariate Protein Signatures of Pre-Clinical Alzheimer's Disease in the Alzheimer's Disease Neuroimaging Initiative (ADNI) Plasma Proteome Dataset
Source: PLoS One. 2012 Apr 2;7(4):e34341. doi: 10.1371/journal.pone.0034341 (PMC3317783; doi:10.1371/journal.pone.0034341)
Supplement: Table S15 — Previous evidence for altered levels of relevant analytes in the context of AD. The table lists the analytes comprising our 11-analyte signatures (Table 3) that have been investigated in the context of MCI or AD. Unless otherwise stated, the referenced studies of plasma/serum or CSF were consistent with the findings in the ADNI plasma samples. Where findings are inconsistent or a literature search returned no information on levels of particular analytes in plasma/serum or CSF, additional indirect evidence has been cited where available from studies of AD brain or other tissues. To our knowledge, the proteins heparin-binding EGF-like growth factor, betacellulin, brain natriuretic peptide and CD5 have not been investigated in the context of AD. (DOC) [file pone.0034341.s020.doc]

Table S15. Previous evidence for altered levels of relevant analytes in the context of AD.

| **Analyte** | **Plasma/Serum** | **CSF** | **Indirect Evidence** |
| --- | --- | --- | --- |
| α2-macroglobulin | ↑ in AD |  | N/A |
| Angiotensinogen |  | ↑ in MCI and AD | N/A |
| Transthyretin | ↓ in AD | ↓ in AD | N/A |
| Serotransferrin | ↓ in AD |  | N/A |
| Apolipoprotein A-II |  | ↓ in AD | N/A |
| Macrophage inflammatory protein-1α | ↓ in AD – inconsistent |  | ↑ in brain microvessels and peripheral T-lymphocytes in AD |
| Glutamic oxaloacetate transaminase |  | ↑ activity in AD | N/A |
| Eotaxin-3 |  | ↑ in cognitive impairment [13] | N/A |
| Fas ligand |  |  | ↑ in neurons of AD brain [14] |
| Peptide YY |  | No change in AD [15] – inconsistent | ↓ peptide YY binding sites in AD brain [16] |

The table lists the analytes comprising our 11-analyte signatures (Table 3) that have been investigated in the context of MCI or AD. Unless otherwise stated, the referenced studies of plasma/serum or CSF were consistent with the findings in the ADNI plasma samples. Where findings are inconsistent or a literature search returned no information on levels of particular analytes in plasma/serum or CSF, additional indirect evidence has been cited where available from studies of AD brain or other tissues. To our knowledge, the proteins heparin-binding EGF-like growth factor, betacellulin, brain natriuretic peptide and CD5 have not been investigated in the context of AD.

**Table References**

1. Hye A, Lynham S, Thambisetty M, Causevic M, Campbell J, et al. (2006) Proteome-based plasma biomarkers for Alzheimer's disease. Brain 129: 3042-3050.

2. Mateos L, Ismail MA, Gil-Bea FJ, Leoni V, Winblad B, et al. (2011) Upregulation of Brain Renin Angiotensin System by 27-Hydroxycholesterol in Alzheimer's Disease. J Alzheimers Dis. Epub ahead of print 2011/02/04.

3. Han SH, Jung ES, Sohn JH, Hong HJ, Hong HS, et al. (2011) Human serum transthyretin levels correlate inversely with Alzheimer's disease. J Alzheimers Dis 25: 77-84.

4. Merched A, Serot JM, Visvikis S, Aguillon D, Faure G, et al. (1998) Apolipoprotein E, transthyretin and actin in the CSF of Alzheimer's patients: relation with the senile plaques and cytoskeleton biochemistry. FEBS Lett 425: 225-228.

5. Gloeckner SF, Meyne F, Wagner F, Heinemann U, Krasnianski A, et al. (2008) Quantitative analysis of transthyretin, tau and amyloid-beta in patients with dementia. J Alzheimers Dis 14: 17-25.

6. Fischer P, Gotz ME, Danielczyk W, Gsell W, Riederer P (1997) Blood transferrin and ferritin in Alzheimer's disease. Life Sci 60: 2273-2278.

7. Zhang J, Goodlett DR, Peskind ER, Quinn JF, Zhou Y, et al. (2005) Quantitative proteomic analysis of age-related changes in human cerebrospinal fluid. Neurobiol Aging 26: 207-227.

8. Zhang J, Sokal I, Peskind ER, Quinn JF, Jankovic J, et al. (2008) CSF multianalyte profile distinguishes Alzheimer and Parkinson diseases. Am J Clin Pathol 129: 526-529.

9. Geppert AM, Losy J, Przedpelska-Ober E, Kozubski W (2010) CCL3 correlates with the number of mood disturbances and personality changes in patients with Alzheimer's disease. Psychiatry Res 176: 261-264.

10. Tripathy D, Thirumangalakudi L, Grammas P (2007) Expression of macrophage inflammatory protein 1-alpha is elevated in Alzheimer's vessels and is regulated by oxidative stress. J Alzheimers Dis 11: 447-455.

11. Man SM, Ma YR, Shang DS, Zhao WD, Li B, et al. (2007) Peripheral T cells overexpress MIP-1alpha to enhance its transendothelial migration in Alzheimer's disease. Neurobiol Aging 28: 485-496.

12. Tapiola T, Lehtovirta M, Pirttila T, Alafuzoff I, Riekkinen P, et al. (1998) Increased aspartate aminotransferase activity in cerebrospinal fluid and Alzheimer's disease. Lancet 352: 287.

13. Craig-Schapiro R, Kuhn M, Xiong C, Pickering EH, Liu J, et al. (2011) Multiplexed immunoassay panel identifies novel CSF biomarkers for Alzheimer's disease diagnosis and prognosis. PLoS One 6: e18850.

14. Su JH, Anderson AJ, Cribbs DH, Tu C, Tong L, et al. (2003) Fas and Fas ligand are associated with neuritic degeneration in the AD brain and participate in beta-amyloid-induced neuronal death. Neurobiol Dis 12: 182-193.

15. Wikkelso C, Ekman R, Westergren I, Johansson B (1991) Neuropeptides in cerebrospinal fluid in normal-pressure hydrocephalus and dementia. Eur Neurol 31: 88-93.

16. Martel JC, Alagar R, Robitaille Y, Quirion R (1990) Neuropeptide Y receptor binding sites in human brain. Possible alteration in Alzheimer's disease. Brain Res 519: 228-235.
